# Aberrant maintenance of developmental transcription factor PAX6 promotes neuronal cell death via JNK3 signaling

**DOI:** 10.1038/s41419-026-08417-6

**Published:** 2026-01-29

**Authors:** Ji-Young Kim, Mi-Jin An, Jinho Kim, Chul-Hong Kim, Yuna Park, Geun-Seup Shin, Hyun-Min Lee, Ah-Ra Jo, Mi Jin Kim, Yujeong Hwangbo, Tae Kyung Hong, Jee Taek Kim, Uimook Choi, Jung-Woong Kim

**Affiliations:** 1 Department of Life Science, Chung-Ang University, Seoul, 06974 South Korea; 2 Ophthalmology Department, College of Medicine, Chung-Ang University, Chung-Ang University Hospital, Seoul, 06974 South Korea; 3 Laboratory of Clinical Immunology and Microbiology, National Institute of Allergy and Infectious Diseases, National Institutes of Health, Bethesda, MD 20892 USA

**Keywords:** Epigenetics, Neural ageing

## Abstract

Retinal ganglion cell (RGC) degeneration is a hallmark of glaucoma and other optic neuropathies, yet the transcriptional mechanisms that drive stress-induced neuronal apoptosis remain incompletely understood. Here, we identify the developmental transcription factor PAX6 as an aberrantly sustained and stress-responsive regulator in mature retinal neurons. Upon NMDA-induced excitotoxic stress, PAX6 is phosphorylated by the neuronal stress kinase JNK3, without changes in total expression levels. In vitro kinase assays confirm direct phosphorylation of PAX6 by JNK3, while genetic ablation of JNK3 abolishes PAX6 activation. This phosphorylation enhances PAX6 chromatin binding and enables its co-recruitment with JNK3 to promoters of pro-apoptotic genes, including *Bax* and *Gadd45a*. Genome-wide ChIP-seq and transcriptomic analyses reveal that PAX6 and JNK3 form a transcriptional complex that drives apoptotic gene expression. In vivo, AAV-shRNA-mediated knockdown of either PAX6 or JNK3 significantly attenuates excitotoxic RGC death. These findings define a previously unrecognized transcriptional mechanism by which JNK3-mediated phosphorylation of persistently expressed PAX6 converts a developmental factor into a driver of neuronal apoptosis. More broadly, this study highlights how the dysregulation of developmental transcriptional programs in postmitotic neurons can contribute to neurodegeneration, offering new mechanistic insights into stress-induced neuronal loss in chronic neurodegenerative diseases.

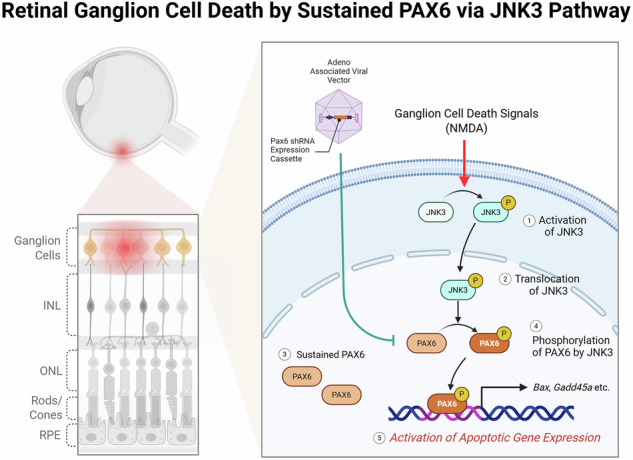

## Introduction

Neurodegeneration in the adult central nervous system (CNS) is often driven by persistent cellular stress and the maladaptive transcriptional reprogramming that follows. Although numerous intracellular signaling cascades have been implicated in this process, the precise molecular pathways linking stress-activated kinase signaling to chromatin-based gene regulation in mature neurons remain incompletely understood. In particular, the molecular switches that control the expression of pro-apoptotic genes in terminally differentiated neurons, such as retinal ganglion cells (RGCs), have yet to be fully elucidated.

Glaucoma is the most common optic neuropathy and a major cause of irreversible blindness, affecting over 80 million individuals globally [1, 2]. The disease is characterized by progressive loss of RGCs and their axons, leading to structural thinning of the neuro-retinal rim and functional deterioration of the visual field. While elevated intraocular pressure (IOP) has long been recognized as a major risk factor, a significant proportion of patients experience disease progression despite well-controlled IOP, suggesting that IOP-independent mechanisms—including oxidative stress, axonal injury, hypoxia, and glutamate-induced excitotoxicity—contribute significantly to RGC death [3, 4]. Indeed, glutamate-mediated NMDA receptor overactivation has emerged as a key pathological mechanism in experimental models of glaucoma, where it drives intracellular calcium overload, mitochondrial dysfunction, and apoptosis in the inner retinal layers, particularly within the ganglion cell layer [5, 6]. However, despite decades of research into glutamate antagonists and neuroprotective small molecules, pharmacological interventions have failed to provide consistent clinical benefit [7, 8], emphasizing the need to understand and directly target the transcriptional regulators that execute cell death programs in RGCs.

Transcription factors involved in early development are increasingly recognized to be repurposed in adult pathophysiology. PAX6, a member of the paired box protein family, is a pivotal transcriptional regulator essential for early eye morphogenesis and neural development in both invertebrates and vertebrates. Canonical *PAX6* and *PAX6(5a)* isoforms orchestrate key processes in retinal neurogenesis, including progenitor proliferation, cell fate specification, and axonal pathfinding [9, 10]. Mutations in PAX6 cause a spectrum of congenital eye diseases, including aniridia and microphthalmia [11–13]. Although PAX6 expression is tightly regulated during embryogenesis, transcriptomic analyses have revealed its unexpected persistence in the adult retina, including RGCs, horizontal cells, and amacrine cells [14, 15]. These findings raise the possibility that PAX6 may function as a latent, stress-inducible transcription factor in fully differentiated retina.

Among stress-responsive signaling cascades, the c-Jun N-terminal kinase (JNK) pathway is one of the most evolutionarily conserved and functionally diverse. The JNK family is encoded by three distinct genes: JNK1 (*Mapk8*), JNK2 (*Mapk9*), and JNK3 (*Mapk10*), with the latter being largely restricted to neuronal tissues [16, 17]. JNKs are activated by a range of cellular insults, including oxidative stress, cytokines, and excitotoxicity, and are known to regulate apoptosis, inflammation, and transcription [18–21]. In the retina, JNK activation has been shown to mediate RGC death through phosphorylation of classical transcription factors such as JUN and p53 [22], and JNK2/3-deficient mice exhibit robust protection against axonal injury and NMDA-induced excitotoxic damage [23]. However, the transcriptional consequences of JNK3 signaling in the mature retina remain poorly defined, particularly in the context of direct regulation of nuclear factors such as PAX6.

Post-translational modifications (PTMs) offer a mechanism through which transcription factors such as PAX6 can acquire context-dependent regulatory functions. Phosphorylation, acetylation, and SUMOylation are known to modulate transcription factor stability, DNA-binding affinity, and cofactor recruitment [24, 25]. PAX6 is known to be acetylated by KAT2A and phosphorylated by MAPKs, including ERK and HIPK2, modifications that fine-tune its transcriptional activity [26–28]. However, whether PAX6 can be dynamically regulated by JNK3 in mature neurons — and whether such regulation impacts pro-apoptotic gene expression — remains entirely unknown.

Together, these findings highlight a potential convergence between stress kinase signaling and developmental transcriptional regulators in the adult retina. In this study, we identify JNK3-dependent phosphorylation of PAX6 as a central mechanism by which stress signaling reprograms the apoptotic gene landscape in mature retina. We show that PAX6 is persistently expressed in RGCs and becomes phosphorylated by JNK3 in response to NMDA-induced excitotoxic stress. Phosphorylated PAX6 displays increased chromatin occupancy at pro-apoptotic loci, including *Bax* and *Gadd45a*, and facilitates the recruitment of JNK3 to these sites, forming a transcriptional complex that drives neuronal apoptosis. Disruption of either PAX6 or JNK3, via genetic knockout (KO) or AAV-shRNA, markedly attenuates RGC degeneration. These findings define a previously unrecognized stress-activated transcriptional circuit in which a developmental transcription factor is repurposed to mediate cell death, offering mechanistic insight into the gene regulatory architecture of neurodegeneration.

## Materials and methods

### Expression strains

C57BL/6 J wild type mice and *Jnk3*^*-/-*^ mice (Strain: #004322) were obtained from the Jackson Laboratory and were maintained as inbred colonies in our animal facility’s pathogen-free, individually ventilated cage housing. Mice were kept in a 12-h light-dark cycle with free access to water and food (lights come off at 20:00). Mice were housed 1-6 per cage and weaned at 3 weeks of age. All animal were approved by the Institutional Review Board of Chung-Ang University (updated IRB no. 2021-00034).

### Bioinformatics processing for scRNA-seq analysis

To profile the cell specific expression patterns in mouse and human retina, we re-analyzed mouse (GSE216694, GSE243413) and human retina scRNA-seq data (GSE196235) [29–31]. For the mouse retina data, we utilized the count matrices from these datasets. To account for technical variations arising from different studies and experiments, we integrated the two datasets and corrected for batch effect using Batch Balanced KNN (BBKNN) algorithm implemented in Scanpy [32]. Following integration, we filtered out mitochondrial and unknown genes, excluded cells with fewer than 200 detected genes, and removed genes expressed in fewer than 3 cells. Subsequently, we performed normalization and log transformation for additional cell-cell normalization. For the human retina scRNA-seq data, we integrated the eight samples from GSE196235 and applied the same preprocessing steps: integration, normalization, and filtering. After preprocessing, we applied the Leiden algorithm to cluster cells, and visualized the clustering results using UMAP in Scanpy [32]. Major cell types were manually annotated based on known cell marker genes.

### Procedure of in vivo intravitreal injection

To establish the NMDA model in mouse, intravitreal injection was performed as previously reported [20]. Intravitreal injections were performed under a microscope with a microsyringe and a 30-gauge micro syringe (Hamilton, Germany) for creating puncture and 22s-gauge of micro syringe for precise delivery, which was inserted ≈1 mm behind the cornea limbus. In control experiments, eyes (*n* = 6 retinas each; 3 independent experiments) were pretreated by injecting 1 μL of 1X PBS (pH 7.4), and in experimental groups, eyes (*n* = 6 retinas each; 3 independent experiments) were treated by injecting 1 μL of 40 mM NMDA (Sigma; corresponding to 40 nmoles) prepared in PBS.

### AAV viral vector production and subretinal injection

The *Pax6* or GFP control was knocked down using AAV viral vectors created and packaged as AAV2.7m8 by Vector Builder (Chicago, USA). For this study, a single batch of AAV2.7m8 vectors was generated, titered at 2.7 to 3.93 × 10^11^ GC/ml, stored in aliquots at 80 °C, and thawed as needed for all experiments to avoid freeze-thawing or potential variations in viral titters. To rule out batch effects due to biological individual differences, we injected each control and knock down AAV construct into the same mouse at the same time. In the left eye, we injected 2 μl of control AAV (pU6-shScramble-GFP AAV2.7m8: 3.87× 10^11^ GC/ml), and in the right eye, we injected 2 μl of knockdown AAV (pU6-shPax6-GFP AAV2.7m8: 3.93 × 10^11^ GC/ml). The retinas were collected separately for IHC two weeks later.

### Immunohistochemistry (IHC)

Mouse retinas or human retinas (IRB no. 2106-006-465) were enucleated and the retinas were excised rapidly by removing the lens in cold PBS. Retinal tissues were fixed with 4% paraformaldehyde (#15710; Electron Microscopy Sciences, Hatfield, PA, USA), and samples were then incubated for 1, 3, and 12 h in solutions containing 10%, 20%, and 30% sucrose-PBS, respectively. The retina was embedded in OCT compound or 7% agarose gel (4583, SAKURA, CA, USA) and then sectioned using a cryotome (CM1850, Leica, Germany) or a vibratome (7000SMZ, Campden Instruments, England). Sections were incubated with 1X sodium citrate buffer (Millipore, 21545) for 10 min at 95 °C for recovering the masked antigen, permeabilized for 10 minutesin PBS containing 0.1% Triton X-100 and then incubated for 1 h in a blocking solution containing 5% normal goat serum in PBS containing 0.1% Triton X-100. Overnight at 4 °C, primary antibodies were incubated. After washing with PBS, secondary antibodies were incubated for 1 h at 23-25 °C in the dark. For 3 min in the dark, the nucleus was counterstained with 5 μg/ml 4’,6-diamidino-2-phenylindole (DAPI). The fluorescence images were created using a Nikon Ti2-E confocal microscope and the NIS-elements BR program (v.5.21.00, Nikon, Japan). Three biological replicates (*n* = 3) were performed for the IHC experiments, and representative images are shown. All antibodies used in this study are provided in Table S9.

### Immunoprecipitation and immunoblot

The retinal tissues were lysed in a solution including 0.1% sodium dodecyl sulfate (SDS), 1% Nonidet P-40, and 1 mM PMSF, 1% Triton X-100, 150 mM NaCl, 50 mM Tris-HCl (pH 7.5). After homogenizing on ice, the cell suspensions from retina tissue were centrifuged at 15,000 g for ten minutes at 4 °C. For immunoprecipitation assays, supernatants were precleaned with 20 μl of protein A/G magnetic agarose beads (50% slurry) for 1 h at 4 °C. Following preclearing, the supernatants were incubated with the appropriate primary antibody overnight at 4 °C with gentle rotation to allow antigen-antibody complex formation. Subsequently, 40 μl of protein A/G magnetic agarose beads were added and incubated for an additional 4 h at 4 °C to capture the immune complexes. The beads were washed three times in PBS before being resuspended in 2X SDS sample buffer and boiled for ten minutes. The protein samples were electrophoresed on an 8-10% SDS-PAGE and transferred to a nitrocellulose membrane (ProtranTM; Whatman, Maidstone, UK). After blocking the membrane with 5% skim milk in TBS-T buffer (137 mM NaCl, 20 mM Tris-HCl, pH 7.6, and 0.1% Tween-20), the primary antibody was diluted appropriately and interacted with the membrane for an overnight at 4 °C. Membranes were washed three times with TBST for 10 min before being incubated for 1 h with a 1:5000 dilution of horseradish peroxidase-conjugated anti-mouse or anti-rabbit antibodies. Following product directions, blots were washed three times using TBST before being developed using the Western blotting luminol reagent (sc-2048, Santa Cruz). Three biological replicates (*n* = 3) were performed, and a representative blot is shown. All antibodies used in this study are provided in Table S9.

### Phosphorylation antibody array

Immunoprecipitates obtained using an anti-PAX6 antibody from mouse retinal lysates were analyzed with a commercial MAPK phosphorylation antibody array (RayBiotech, Norcross, GA, USA) according to the manufacturer’s instructions. Briefly, the membranes were blocked with blocking buffer for 30 min at room temperature, followed by an overnight incubation at 4 °C with PAX6 (Abcam, Cat ab5790) immunoprecipitants diluted 1:2 in blocking buffer. After washing steps, an antibody cocktail was applied and incubated overnight at 4 °C. This was followed by a further 2 h incubation at 4 °C with HRP-Conjugated anti-IgG at room temperature. The membranes were then treated with a peroxidase substrate and the signals were detected by the chemiluminescence imaging system. Chemiluminescence signal intensity was quantified using ImageJ software.

### Total RNA isolation and RT-qPCR

Following the manufacturer’s instructions, total RNA was extracted using the TRIzol solution (15596018, Invitrogen, CA, USA). By mixing 20 units of Rnase-free Dnase I (New England Biolabs) and 4 units of RNase inhibitor (New England Biolabs) with DEPC-treated water, contaminated genomic DNA was eliminated from 10 μg of total RNA. The reaction mixture was incubated for 10 min at 50 °C after incubation for 1 h at 37 °C. All RNA extracts had an OD260:OD280 ratio between 1.8 and 2.0, indicating high purity of the RNA. Oligo-dT (6110 A, Takara) was applied as the primer in the first step of cDNA synthesis. Total RNA (500 ng) was mixed with 1 μl of oligo dT, and distilled water and then preheated at 65 °C for 5 min to denature the secondary structures of RNA. The mixture was quickly cooled to 4 °C, before adding 10 mM DTT, 4 μl of 5X PrimeScript buffer, and 200 units of PrimeScript reverse transcriptase (#RR036A, PrimeScript™ RT Master Mix, Takara) to make a total volume of 20 μl. The reverse-transcriptase mixture was incubated at 25 °C for 5 min then incubated at 42 °C for 60 min before being stopped by heating at 70 °C for 15 min. The cDNA stock was stored at -20 °C. According to the manufacturer’s instructions, the CFX96 Real-time PCR detection equipment (Bio-Rad) and the iQ SYBR Green PCR Supermix (#1708880, Bio-Rad) were used to detect amplified cDNA samples for real-time quantitative PCR. Melting curve analysis was used to demonstrate the uniqueness of each amplified product. The -actin gene was used as for normalization. The relative mRNA expression was calculated by the 2^-(ΔΔCt)^ method.

### RNA sequencing and bioinformatical analysis

According to the manufacturer’s instructions, the TruSeq mRNA Library Prep Kit (Illumina, Inc., USA) generated the RNA-seq library. To be brief, 100 ng of total RNA from retinas (> 3 retinas) were extracted, and then reverse transcription was carried out using an oligo-dT primer that had an Illumina-compatible sequence at its 5’ end. After degradation of the RNA template, a random primer with an Illumina-compatible linker sequence at its 5’ end started the second strand synthesis. The AMPure magnetic beads (A63881, Beckman Coulter, CA, USA) were used to remove all reaction components from the double-stranded library during purification. The library was amplified to include all of the adapter sequences required for cluster generation. The finished library was purified from PCR components. High-throughput sequencing was carried out as paired-end 101 sequencing reads using NextSeq 500 (Illumina, Inc., USA). STAR-2.7.1a was used to align mRNA-Seq reads. Indices were generated from either the genome assembly sequence or representative transcript sequences for alignment to the genome and transcriptome (GRCm38.p6.101). The alignment file was used to assemble transcripts, estimate their abundances, and detect gene expression differences using RSEM (v.1.3.3). Differentially expressed transcripts were identified using EdgeR (v.3.36.0) within R version 4.1.0 (R development Core Team, 2021) using the R package.

### Production of recombinant protein

The protein sequence corresponding to amino acid 1-422 of mouse PAX6 and 1-464 of mouse JNK3 were amplified by PCR and subcloned into the pGEX-4T3 vector. E. coli BL21 (DE3) cells with plasmid were grown at 37 °C in LB medium. Protein expression was induced with 1 mM IPTG at an OD600 of ~0.6 and incubated for an overnight at 30 °C. The cells were harvested via centrifugation and resuspended in ice cold lysis buffer containing 20 mM Tris (pH 8.0), 0.1 M NaCl, 1 mM EDTA, 1X proteinase inhibitor cocktail, and 1 mM PMSF. The cells were lysed using a sonicator and centrifuged at 15,000 g for ten minutes at 4 °C. The supernatant was incubated with glutathione sepharose 4B resin (GE Healthcare Life Sciences) for 3 h and washed with the wash buffer (same as lysis buffer containing 0.5% IGEPAL CA-630) three times, and eluted with elution buffer (50 mM Tris (pH 8), 0.15 M NaCl, 100 mM reduced glutathione) for 20 min at 4 °C. Eluted GST-PAX6 and GST-JNK3 were used for kinase assays.

### In vitro kinase assay

Eluted GST-PAX6 and GST-JNK3 were mixed and incubated with 5 μCi of [γ-^32^P] ATP (Perkin Elmer, Waltham, MA) or 20 μM ATP (A2383; Sigma-Aldrich) in 30 μl of kinase buffer (25 mM HEPES (pH 7.6), 20 mM MgC1_2_, 20 mM β-glycerophosphate (G6251, Sigma-Aldrich), 0.1 mM sodium orthovanadate (S6508, Sigma-Aldrich), 2 mM dithiothreitol (D0632, Sigma-Aldrich)) for 30 min at 30 °C. The kinase reactions were terminated by the addition of 2X protein loading dye and boiled for 10 min Reaction products were separated by SDS-PAGE and analyzed by autoradiography.

### Chromatin immunoprecipitation (ChIP) assay

Retinal tissue samples were cross-linked with 1% paraformaldehyde for 10 min at 23 °C and quenched with glycine at a final concentration of 125 mM. The samples were homogenized in SDS lysis buffer (1% SDS, 10 mM EDTA, and 50 mM TrisHCl (pH 8.1)), and chromatin was sheared using a bioruptor sonicator (Diagenode) at high power for 45 cycles (30 s on / 30 s off, repeat twice). After centrifugation for 10 min at 18,500 g, samples were resuspended in ChIP dilution buffer (0.01% SDS, 1.2 mM EDTA, 1.1% Triton X-100, 167 mM NaCl, and 16.7 mM Tris-HCl (pH 8.1)). Immunoprecipitation was performed overnight on the sonicated lysates using the designated antibodies (5 μg of antibody per IP reaction). Protein A/G magnetic beads (26162, Thermo Scientific, USA) were added, and immunoprecipitations were continued for an additional 4 h. After that, the samples were washed using low-salt wash buffer (0.1% SDS, 1% Triton X-100, 2 mM EDTA, 150 mM NaCl, 20 mM Tris-HCl (pH 8.1)), high-salt wash buffer 0.1% (1 mM EDTA, 10 mM Tris-HCl (pH 8.0)), LiCl immune-wash buffer (0.25 M LiCl, 1% NP40, 1% deoxycholate, 1 mM EDTA, 10 mM Tris-HCl (pH 8.1)), and Tris-EDTA buffer (1 mM EDTA, 10 mM Tris-HCl (pH 8.0)). After the last wash, the immunoprecipitates were eluted twice with 100 μl of IP elution buffer (1% SDS, 0.1 M NaHCO_3_) and the samples were reverse-crosslinked at 65 °C for an overnight. DNA/protein complexes were precipitated with 100% ethanol, air-dried, and dissolved in 20 μl of distilled water (W4502, Sigma). The list of primers for validation of ChIP-seq was described in Table S10.

### ChIP-seq library preparation and bioinformatical analysis

The TruSeq ChIP Library Preparation Kit (Set A, IP-202-1012, Set B, IP-202-1024, Illumina, CA, USA) was used to create the ChIP-seq library. To summarize, 5 μg of input and ChIP-enriched DNA were end-repaired, A-tailed, and ligated with TruSeq index adapters before being amplified. The Illumina NextSeq 500 platform was used for paired-end sequencing of all ChIP libraries. All ChIP reads in FASTQ format were aligned to the GRCm38.p6.101 mouse genome using Bowtie2 (v 2.4.4) and removed redundant reads. Peak calling was carried out using MACS2 (ver 2.2.6) with “–nomodel -f AUTO -p 0.001” options. After confirming the consistency between each replicate, we pooled extended reads and generated the bigwig track for visualization with IGV (ver.2.8.7).

### Gene ontology and gene set enrichment analysis (GSEA) analysis

Significant DEGs and ChIP-seq peaks in respective gene sets were clustered into functional gene ontologies using DAVID (http://david.abcc.ncifcrf.gov). Metascape was used to identify enriched gene ontology terms, and REVIGO was used to create scatter plots of ontology terms. GSEA analysis was carried out using (GSEA 4.1.0; http://www.broadinstitute.org/gsea/index.jsp) and the MsigDB v7.1 mouse database.

### Image analysis

The number of GCLs was counted in the middle of *Jnk3*^*+/+*^ or *Jnk3*^*-/-*^ mice inferior retina. Intensities of cytochrome c were measured within each cell’s cytoplasm by using ‘automated measurement’ plugin NIS software.

### Statistical analysis

GraphPad PRISM software was used to examine the significance of differences in result data using the Student’s t-test and Mann-Whitney U test, as appropriate. The results were described using the mean standard error of the mean (SEM) and were obtained from two or three separate experiments. Statistically significant *P* values were less than 0.05.

## Results

### PAX6 is selectively retained in mature RGC of both mouse and human retinas

**Fig. 1:**
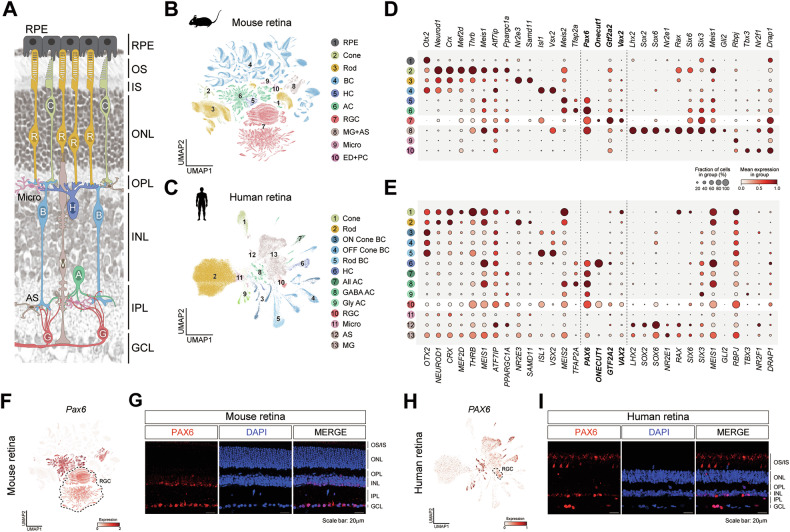
PAX6 is persistently expressed in RGCs of adult mouse and human retinas. **A** Schematic representation of neural retina structure. A, amacrine cells; B, bipolar cells; C, cone photoreceptors; G, ganglion cells; H, horizontal cells; M, Müller glia; R, rod photoreceptors. **B** t-SNE visualization of various cell types in mouse retinas, indicated using different colors and annotated into 10 transcriptionally distinct clusters (1–10 groups) based on scRNA-seq. **C** t-SNE visualization of various cell types human retinas, indicated using different colors and annotation into 13 distinct clusters (1–13 groups) based on scRNA-seq. **D, E** Feature expression heatmap showing transcription factors in the retina of mouse **D** and human **E**. **F, H** t-SNE visualization of the expression patterns of *Pax6* in mouse **F** and human **H** retinas. **G, I** Immunostaining of PAX6 (red) and DAPI in mouse **G** and human **I** retinas. Scale bar: 20 μm. The PAX6 signals in **I** observed in the outer segments of the human retina are non-specific, as PAX6 is not known to be expressed in photoreceptor outer segments, and these signals are likely result from non-specific antibody background or tissue autofluorescence.

Transcription factors that orchestrate retinal development are increasingly recognized to have regulatory roles beyond neurogenesis, yet their expression dynamics in adult retina remain incompletely characterized. To identify developmental transcription factors retained in the mature retina, we reanalyzed publicly available single-cell RNA-seq (scRNA-seq) datasets from adult human (GSE196235) and mouse retinal tissues (GSE216694, GSE243413) [29–31]. Dimensional reduction and clustering of the datasets revealed transcriptionally distinct populations corresponding to RGCs, amacrine, bipolar, and horizontal cells (Fig. 1A–C and Fig. S1A, B). Among the most persistently expressed developmental regulators, PAX6 exhibited high and selective expression in RGCs, horizontal, and amacrine cells across both species (Fig. 1D, E). Visualization of PAX6 transcript abundance and histological validation confirmed strong PAX6 expression in the ganglion cell layer (GCL) and inner nuclear layer (INL), where RGCs and amacrine cells reside (Fig. 1F–I). The GCL of the retina contains both RGCs and displaced amacrine cells in relatively similar proportions [33, 34]. To further clarify the cellular specificity of PAX6 expression in the GCL, we performed co-immunostaining with cell-type-specific markers. PAX6 showed minimal overlap with Calb1^+^ or ChAT^+^ amacrine cells, whereas it strongly co-localized with Brn3a^+^ RGCs (Fig. S1C). These results demonstrate that PAX6 expression in the GCL is largely confined to RGCs rather than displaced amacrine cells. Importantly, PAX6 expression is not developmentally silenced but maintained in fully differentiated retina, particularly RGCs, suggesting a potential functional role in adult retinal gene regulation and a mechanistic link to stress-induced transcriptional control in disease contexts such as glaucoma.

### PAX6 regulates pro-apoptotic gene expression in NMDA-induced RGC degeneration

**Fig. 2:**
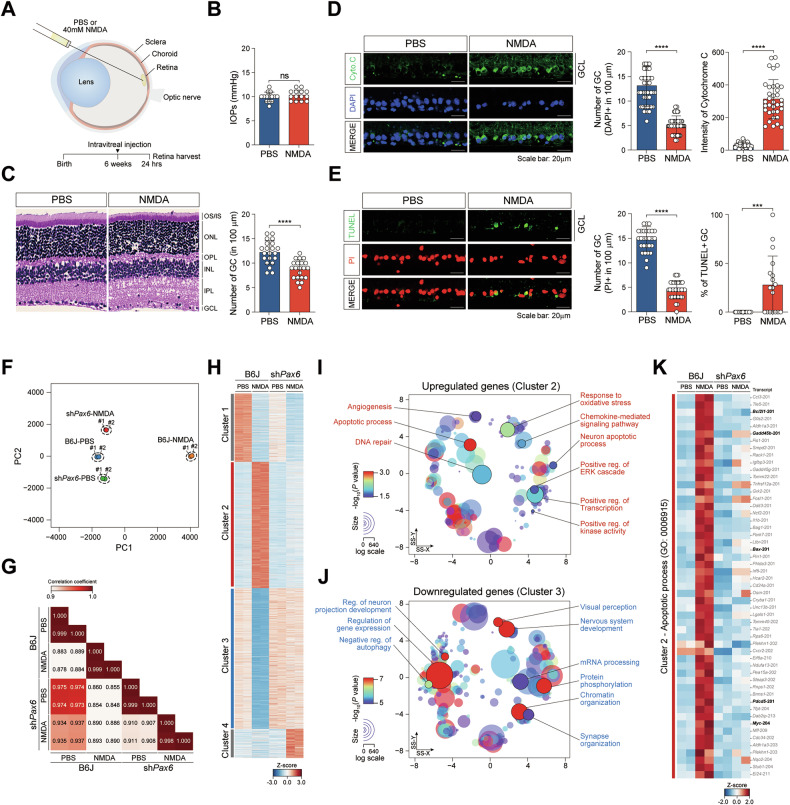
PAX6 regulates apoptotic genes in mouse retina following NMDA-induced excitotoxicity. **A** Outline of the experimental procedure for NMDA administration via intravitreal injection. **B** The bar chart represents changes in IOP in both PBS and NMDA-treated mouse retinas. **C** NMDA- and PBS-treated retinas were stained with DAPI and H&E (left); scale bar: 20 μm. Quantification of the number of GC on 100 μm in PBS and NMDA-excitotoxicity mouse retinas (right). **D** Immunostaining of cytochrome c in PBS- and NMDA-treated mouse retinas (left); scale bar: 20 μm. The number of GC and cytochrome c intensity in PBS- and NMDA-treated mouse retinas was measured using ImageJ (right). **E** Apoptotic cells in PBS- and NMDA-treated mouse retinas were detected using TUNEL assay (BrdU positive cells); scale bar: 20 μm. Number of GC and BrdU-positive GC in PBS- and NMDA-treated mouse retinas were measured using ImageJ (right). Each quantification graph includes *n* = 10 per group, representing biologically independent retinas from three different animals. All error bars represent SEM. *P* values obtained by Student’s t-test. **** *P* < 0.0001, *** *P* < 0.001, n.s., not significant. **F** PCA plot of RNA-seq data of the retina of a 2-month-old WT mouse intravitreally injected with sh*Pax6*-GFP AAV2.7m8. **G** Correlation matrix of Pearson correlation coefficient values for each indicated sample set. Shades of red indicate an increasing positive correlation coefficient. Correlation values are shown with three-decimal precision to accurately reflect the measured correlations. **H** A hierarchical clustering heatmap was generated to display genes that showed differential expressions. **I, J** A scatter plot illustrating the confidence scores for enriched gene ontologies linked to upregulated **I** and downregulated genes **J**. SS-X, semantic space X, and SS-Y, semantic space Y. **K** Heatmap showing DEGs (in PBS- or NMDA-injected) mapped to apoptotic processes in control (B6J) and sh*Pax6*-GFP AAV2.7m8 infected (sh*Pax6*) mouse retinas.

To directly investigate whether persistent PAX6 expression contributes to RGC vulnerability under stress, we employed a glutamate excitotoxicity model using intravitreal NMDA injection in adult mice, a well-established approach to mimic excitotoxic neuronal injury in RGCs through overactivation of NMDA-type glutamate receptors [35]. To assess the effects of excitotoxic stress in vivo, we intravitreally injected 40 mM NMDA (1 µL; *n* = 4) or phosphate-buffered saline (PBS, used as a control, 1 µL; *n* = 4) into adult WT mouse eyes (Fig. 2A). IOP remained unchanged across treatment groups, ruling out pressure-dependent effects (Fig. 2B)[36]. Hematoxylin and eosin staining revealed a selective reduction in the number of RGCs in NMDA-treated eyes compared to controls, while the outer and inner nuclear layers remained morphologically intact (Fig. 2C). This selective vulnerability of RGCs was further supported by whole-mount immunostaining showing decreased RGC density following NMDA injection (Fig. S2A, B).

We first assessed apoptotic signaling in RGCs by examining cytochrome c release, a mitochondrial marker of apoptosis. Immunofluorescence showed significantly increased cytochrome c intensity localized within the GCL of NMDA-injected retinas (Fig. 2D and Fig. S2C). In parallel, TUNEL staining revealed a marked increase in BrdU-positive apoptotic nuclei specifically within RGCs, whereas amacrine and horizontal cells in the INL remained unaffected (Fig. 2E and Fig. S2D). These data confirm that NMDA administration induces selective RGC apoptosis in vivo, recapitulating a key aspect of glaucoma-associated neurodegeneration. To further determine whether apoptosis in the GCL also involves displaced amacrine cells, we performed co-immunostaining for cytochrome c with the amacrine marker ChAT. In PBS-treated retinas, cytochrome c release was rarely detected in either ChAT^+^ or ChAT^-^ cells. In contrast, NMDA treatment induced robust cytochrome c release predominantly in ChAT^-^ cells, which based on Brn3a staining corresponds to RGCs, while ChAT^+^ amacrine cells showed only minimal positivity (Fig. S2E). Quantification confirmed that most apoptotic cells were ChAT^-^/cytochrome c^+^ RGCs (~57%), whereas ChAT^+^/cytochrome c^+^ amacrine cells accounted for only a minor fraction (~16%) (Fig. S2F). These results demonstrate that NMDA-induced excitotoxic apoptosis in the GCL is predominantly confined to RGCs, with displaced amacrine cells contributing only minimally. To further explore the contribution of PAX6 to the excitotoxic gene regulatory response, we performed functional knockdown using AAV2.7m8 carrying shRNA targeting *Pax6* (sh*Pax6*-GFP), which was intravitreally delivered into 2-month-old mice (Fig. S3A, B). Efficient PAX6 knockdown in the retina, particularly in RGCs, was confirmed by immunostaining three weeks post-injection, with strong GFP reporter expression indicating successful AAV transduction (Fig. S3C). In contrast, retinas injected with a control shScramble-GFP vector maintained normal levels of PAX6 expression.

We next performed transcriptome-wide RNA sequencing on retinas from B6J and sh*PAX6*-infected mice following intravitreal infection of either PBS or NMDA (Table S1). Principal component analysis (PCA) revealed that NMDA-treated control retinas clustered separately from both PBS-treated and PAX6 knockdown samples, indicating robust NMDA-dependent transcriptional reprogramming and its attenuation by PAX6 silencing (Fig. 2F). Pearson correlation analysis further confirmed high reproducibility between biological replicates (Fig. 2G). To provide an overview of global transcriptomic alterations, we generated volcano plots comparing PBS- and NMDA treated retinas in both B6J and sh*Pax6*-infected mice. These plots show the statistical significance of differentially expressed transcripts (DETs) relative to their respective fold changes (*p* < 0.05, absolute log_2_ fold-change > 1, and variance-mean ratios < 2) compared with the control group, thereby highlighting condition-specific gene expression shifts (Fig. S3D, E). Differential expression analysis based on these criteria revealed 1,185 DETs specifically altered in the NMDA-treated sh*Pax6* conditions. These transcripts were subsequently categorized into four primary expression clusters via k-means clustering (Fig. 2H and Fig. S3F and Tables S2, 3). Additionally, Gene ontology (GO) analysis of upregulated genes (cluster 2) in control NMDA-treated retinas showed enrichment for apoptosis, calcium signaling, and NF-κB–mediated stress responses (Fig. 2I and Table S4). In contrast, genes downregulated by PAX6 knockdown (cluster 3) were associated with chromatin regulation, protein trafficking, and DNA repair (Fig. 2J and Table S4), consistent with a broad regulatory role for PAX6 in both injury responses and transcriptional homeostasis. Critically, heatmaps of apoptotic pathway genes revealed strong induction of mitochondrial- and caspase-mediated apoptosis pathways in NMDA-injured retinas, which were significantly suppressed in PAX6-knockdown eyes (Fig. 2K and Fig. S3G and Table S5).

### NMDA-induced excitotoxicity enhances JNK3–PAX6 interaction in RGCs

**Fig. 3:**
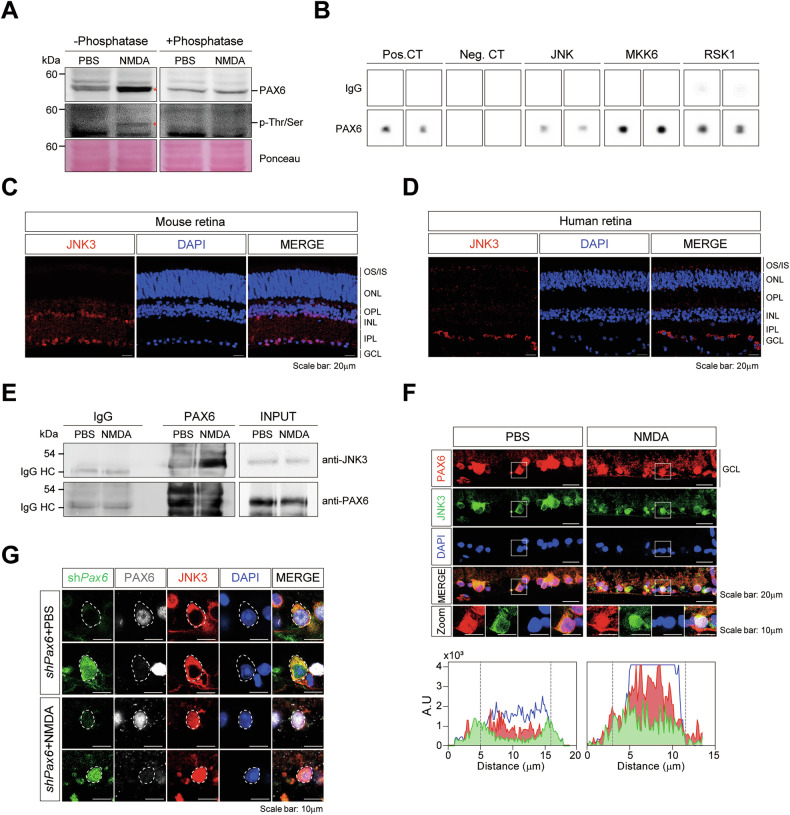
PAX6 physically interacts with JNK3 in response to excitotoxic injury mouse retinas. **A** PBS- or NMDA-injected mouse retinas were treated with lysis buffer containing 20 μM of phosphatase for 30 min and immunoblotted with anti-PAX6 and anti-p-Thr/Ser antibodies. The asterisks indicate the phosphorylated form of PAX6 detected under NMDA-treated conditions. Three biological replicates were performed, and a representative blot is shown. **B** Profiles of PAX6 binding to the kinases in the human phosphorylation antibody array. The increase corresponds to the binding of PAX6 to JNK, MKK6, and RSK1, respectively. **C, D** Immunostaining for JNK3 (red) and DAPI in mouse **C** and human **D** retinas. Scale bar: 20 μm. **E** Immunoblot showing co-immunoprecipitation of PAX6 with JNK3 in PBS- and NMDA-treated mouse retinas. Goat serum IgG was used as a negative control for the co-immunoprecipitation assays. Three biological replicates (*n* = 3) were performed, and a representative blot is shown. **F** Immunostaining with PAX6 (red), JNK3 (green), and DAPI (blue) in PBS- and NMDA-treated mouse retinas. Scale bar: 20 μm. Higher magnification images in the white box indicate PAX6 (red), JNK3 (green), and DAPI (blue) staining in ganglion cells. Scale bar: 10 μm. The quantification of each immunostaining intensity in the nucleus of ganglion cells. **G** Immunostaining with PAX6 (white), JNK3 (red), and DAPI (blue) staining in RGCs infected with sh*Pax6*-GFP AAV2.7m8, following intravitreal injection of PBS or NMDA. Green fluorescence indicates sh*Pax6*-driven GFP expression. Scale bar: 10 μm.

To explore upstream mechanisms regulating PAX6 activity in the context of NMDA-induced retinal injury, we investigated whether this activation was associated with altered PAX6 expression or post-translational modification following excitotoxic stress. Notably, immunohistochemistry showed that PAX6 protein expression in the GCL remained unchanged after NMDA administration (Fig. S4A), suggesting that post-translational modifications rather than transcriptional induction may underlie PAX6 activation. Consistently, although the PAX6 band appeared stronger in NMDA-treated samples under non-dephosphorylated (-phosphatase) conditions, this difference was abolished following phosphatase treatment, indicating that the apparent increase was due to phosphorylation rather than an actual increase in total protein levels. Transcription factors such as PAX6 are known to interact with coregulators, including kinases that modulate their activity through phosphorylation. Indeed, previous studies have shown that PAX6 can be phosphorylated by kinases such as p38 and HIPK2 to regulate its transcriptional function [27, 28]. To assess whether NMDA exposure induces PAX6 phosphorylation in vivo, we performed Western blotting with phospho-threonine/serine antibodies and observed a significant increase in phosphorylated PAX6 in NMDA-injected WT retinas compared to controls (Fig. 3A), implicating stress-activated kinase involvement.

To identify candidate kinases responsible for this phosphorylation, we conducted an antibody-based phospho-kinase interaction screen. PAX6 was found to interact with several MAPK pathway components, including JNK1 (*Mapk8*), JNK2 (*Mapk9*), JNK3 (*Mapk10*), MEK1 (*Map2k1*), MKK6 (*Map2k6*), and RSK1 (*Rps6ka1*) (Fig. 3B and Fig. S4B). Among these, JNK3 (*Mapk10*) emerged as a strong candidate based on both protein interaction and expression data. scRNA-seq analysis and immunostaining confirmed that JNK3 is highly expressed in RGCs of both mouse and human retinas (Fig. 3C, D and Fig. S4C–E), in contrast to the broader expression of JNK1 and JNK2.

We next tested whether PAX6 physically associates with JNK isoforms in the retina. Co-immunoprecipitation using anti-PAX6 antibodies revealed interactions with JNK1, JNK2, and JNK3 in NMDA-injected retinal lysates, with JNK3 displaying the strongest binding signal among the isoforms (Fig. S4F). To evaluate spatial localization, we performed IHC for PAX6 and each JNK isoform in PBS- or NMDA-treated retinas (Fig. S4G). NMDA-induced excitotoxicity led to prominent co-localization of PAX6 with JNK3 in the GCL, whereas overlap with JNK1 or JNK2 remained minimal, supporting a preferential interaction between PAX6 and JNK3 under excitotoxic conditions. Focusing on JNK3, we confirmed enhanced interaction with endogenous PAX6 following NMDA treatment, as shown by reciprocal IP and immunoblotting (Fig. 3E). Notably, while total JNK3 protein levels were comparable between conditions, the amount of JNK3 co-immunoprecipitated with PAX6 increased markedly under NMDA stress. These findings suggest that NMDA-induced excitotoxicity promotes the formation or stabilization of the JNK3-PAX6 complex, likely through JNK3 activation and nuclear translocation. Furthermore, immunohistochemical analysis revealed increased nuclear localization of JNK3 in the GCL of NMDA-injected retinas, overlapping with PAX6 expression (Fig. 3F). To test whether PAX6 is required for JNK3 nuclear translocation, we performed similar staining in sh*Pax6*-GFP AAV-infected retinas and observed that JNK3 nuclear entry occurred independently of PAX6 (Fig. 3G and Fig. S4H). This suggests that JNK3 acts upstream of PAX6, and that nuclear co-localization is a consequence of JNK3 activation under stress. Collectively, these data demonstrate that NMDA-induced excitotoxicity promotes the physical and spatial association between PAX6 and JNK3 in RGCs, supporting a model in which JNK3 phosphorylates PAX6 to modulate its transcriptional activity during retinal neurodegeneration.

### JNK3 directly phosphorylates PAX6 in vitro and in vivo following NMDA-induced excitotoxicity

**Fig. 4:**
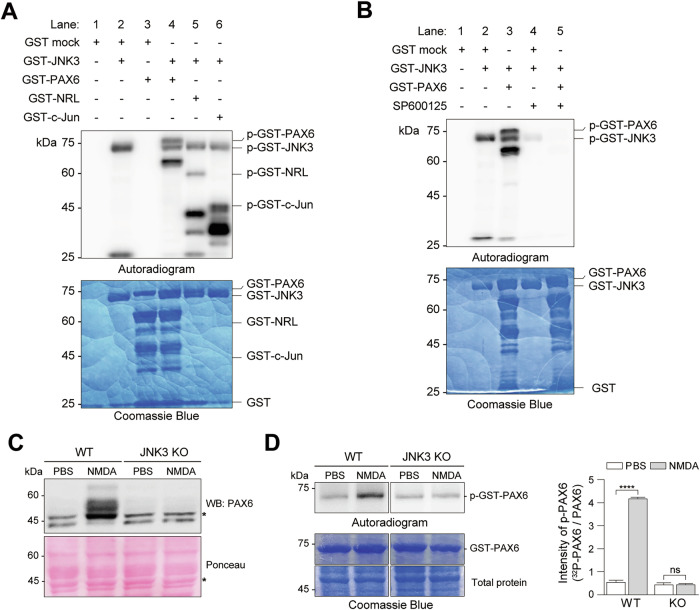
JNK3 directly phosphorylates PAX6 both in vitro and in vivo following excitotoxic stress. **A** Purified GST-PAX6 was incubated with GST-JNK3 30 mins at 30 °C. Reaction mixtures were separated using SDS-PAGE and analyzed via an autoradiography. GST-NRL and GST-c-Jun were used as positive controls, while GST alone was the negative control. **B** Kinase assay procedure **A**, GST-PAX6 and GST-JNK3 were pretreated with 100 μM of the JNK inhibitor SP600125. **C** Whole retinal lysates of PBS- and NMDA-treated WT and JNK3 KO mice were immunoblotted using an anti-PAX6 antibody. Ponceau staining was used for internal loading control. The asterisks indicate the phosphorylated forms of PAX6 under NMDA-treated conditions. Three biological replicates were performed, and a representative blot is shown. **D** Whole retinas of PBS or NMDA-treated WT and JNK3 KO mice were lysed with kinase assay lysis buffer containing phosphatase inhibitors and incubated with GST-PAX6 fusion proteins. The phosphorylated proteins were resolved using SDS-PAGE and detected via autoradiography (left). Signal intensity of p-PAX6 was quantified using ImageJ software (right). All error bars represent SEM. *P* values were obtained using Student’s t-test. **** *P* < 0.0001, n.s., not significant.

Given that PAX6 contains multiple serine and threonine residues within its transactivation domain (TAD) [37], we tested whether it could serve as a substrate for phosphorylation by JNK3, a neuronal stress kinase. To examine whether JNK3 activation induces PAX6 phosphorylation in vitro, we conducted a kinase assay using bacterially expressed and purified GST-fusion proteins. GST-JNK3 was added to the kinase assay along with GST-PAX6 and control substrates (GST, GST-NRL, and GST-c-Jun) in the presence of [γ-^32^P] ATP isotope (Fig. 4A and Fig. S5A). GST-JNK3 strongly phosphorylated GST-PAX6, while GST alone was not phosphorylated, confirming the specificity of the reaction (Fig. 4A, lane 4 and Fig. S5A, lane 4). Known JNK3 substrates, GST-NRL and GST–c-Jun, served as positive controls and showed robust phosphorylation under the same conditions (Fig. 4A, lanes 5–6). As expected, we also observed autophosphorylation of GST-JNK3, consistent with its intrinsic activation mechanism (Fig. 4A, lane 2 and Fig. S5A, lane 2) [38]. To validate this, immunoprecipitation of retinal lysates confirmed enhanced phosphorylation of JNK3 following NMDA-induced injury (Fig. S5B), supporting the notion that JNK3 is autoactivated in vivo under excitotoxic conditions.

To further assess whether JNK3 directly phosphorylates PAX6 in vitro, we performed a kinase assay with GST-PAX6 under increasing concentrations of GST-JNK3. Importantly, activated JNK3 enhanced PAX6 phosphorylation in a dose-dependent manner (Fig. S5C). Additionally, we investigated whether the JNK inhibitor SP600125 can inhibit JNK3-induced phosphorylation of PAX6. SP600125 significantly suppressed both JNK3 autophosphorylation and GST-PAX6 phosphorylation (Fig. 4B, lanes 4-5), and this inhibition was dose-dependent (Fig. S5D), indicating that the observed phosphorylation events were JNK activity–dependent. We next examined whether PAX6 phosphorylation occurs in vivo and whether this modification is JNK3-dependent. Immunoblotting of retinal lysates from WT mice revealed multiple phosphorylated PAX6 bands following NMDA treatment, whereas JNK3 KO mice failed to show comparable phosphorylation levels (Fig. 4C). These findings indicate that JNK3 is necessary for PAX6 phosphorylation in vivo during retinal injury. To corroborate these results, we performed a GST-PAX6 in vitro kinase assay using lysates from NMDA- or PBS-injected retinas of WT and JNK3 KO mice. GST-PAX6 phosphorylation was robustly increased in lysates from NMDA-treated WT mice, but not in those from NMDA-treated JNK3 KO mice, or PBS controls (Fig. 4D). The absence of phosphorylation in JNK3-deficient samples confirms that PAX6 phosphorylation in the injured retina requires JNK3 activity. Together, these results provide strong biochemical and genetic evidence that JNK3 directly phosphorylates PAX6 under excitotoxic stress, both in vitro and in vivo, establishing a mechanistic link between JNK3 activation and transcriptional modulation via PAX6 during retinal neurodegeneration.

### PAX6 and JNK3 cooperatively regulate pro-apoptotic transcriptional programs via chromatin occupancy in excitotoxic retinas

**Fig. 5:**
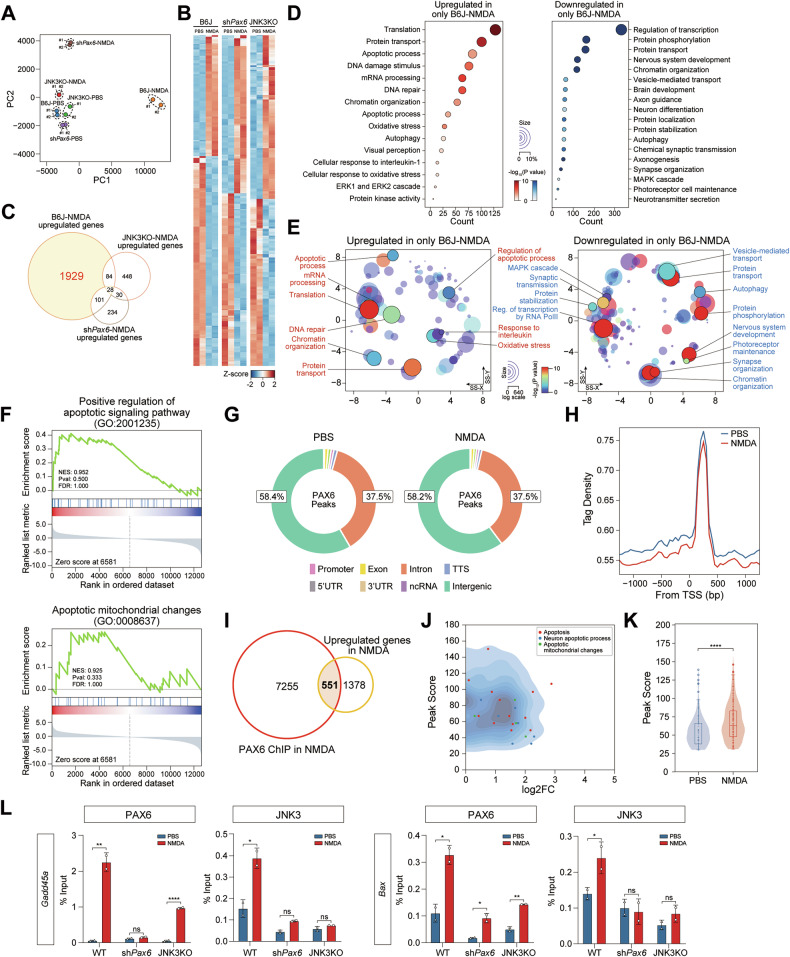
PAX6 and JNK3 cooperatively regulate pro-apoptotic gene expression through chromatin engagement at target loci. **A** PCA plot of RNA-seq data of the retina of WT, JNK3 KO, and sh*Pax6*-GFP AAV2.7m8 mice. **B** Hierarchical clustering heatmap was generated to display the expression profiles of genes. **C** Venn diagram showing the overlap of DEGs among the three comparisons. **D** Gene ontology (GO) analysis of upregulated and downregulated genes among the three comparisons using DAVID tool. **E** GO enrichment analysis summarized and visualized as a scatter plot using REVIGO. GO terms are represented by circles and are plotted based on their semantic similarity to other GO phrases (circles that are adjacent represent the closest relationships). The size of the circle is directly related to the frequency of the GO term, whereas color indicates the log10 *P* value. SS-X, semantic space X, and SS-Y, semantic space Y. **F** GSEA scores for genes involved in positive regulation of the apoptotic signaling pathway and apoptotic mitochondrial changes. **G** Pie chart of PAX6 enrichment distribution at genomic loci in 2-month-old PBS or NMDA-injected mouse retinas. **H** Histogram shows the distribution of PAX6 peaks around transcriptional start sites (± 1 Kb) in PBS or NMDA-injected mouse retinas. **I** Venn diagram showing the overlap between the genes, including PAX6 peaks and upregulated PAX6 and JNK3 target genes in the retinas of mice with NMDA-induced excitotoxicity based on RNA-seq data. **J** Scatter plot showing changes in the expression of apoptotic genes, neuron apoptotic process, and apoptotic mitochondrial changes in the retinas of mice with NMDA-induced excitotoxicity and their enrichments for PAX6. **K** Violin plot shows the score of PAX6 peaks around upregulated genes in NMDA-injected mouse retinas. *P* values obtained by Mann-Whitney U test. **** *P* value < 0.0001. **L** Recruitment and presence of PAX6 and JNK3 at *Gadd45a* and *Bax* promoter regions are validated using ChIP-qPCR. Error bars show mean ± SE. *P* values based on Student’s *t*-test. **** *P* value < 0.0001, ** *P* value < 0.01, * *P* value < 0.05, n.s., not significant.

To understand the transcriptional consequences of JNK3–PAX6 signaling in the injured retina, we performed bulk RNA sequencing of retinas from WT, AAV2.7m8-sh*Pax6*-infected, and JNK3 KO mice following NMDA or PBS injection. PCA demonstrated robust transcriptomic divergence in NMDA-treated WT retinas compared to controls, highlighting the global transcriptional response to excitotoxic stress (Fig. 5A). Pearson correlation analysis further supported the reproducibility and condition-specificity of the transcriptomic profiles, showing reduced similarity between WT and both Pax6 knockdown and JNK3 KO groups (Fig. S6A).

Differential expression analysis identified 6,371 transcripts with altered expression across groups, based on fragments per kilobase of transcript per million mapped reads (FPKM) > 1 in at least one group and *p* < 0.05, with 853 and 1,264 DETs being unique to the sh*Pax6* and JNK3 KO conditions, respectively (Fig. 5B and Fig. S6B; Table S2). Notably, 1,929 genes were significantly upregulated in NMDA-treated WT retinas but were either downregulated or unchanged in sh*Pax6* or JNK3 KO retinas, suggesting that their expression is co-regulated by both factors (Fig. 5C). GO and GSEA of these NMDA-induced transcripts revealed strong enrichment for apoptotic processes, mitochondrial disruption, oxidative stress, and DNA damage response pathways (Fig. 5D–F and Table S6). These transcriptional signatures were significantly attenuated in both *Pax6* knockdown and JNK3-deficient conditions, implicating both proteins as central regulators of excitotoxic gene activation.

To further elucidate the role of PAX6 in gene regulation in NMDA-induced excitotoxicity, we performed a chromatin immunoprecipitation assay of NMDA-treated retinas of WT mice followed by deep sequencing (ChIP-Seq) using an anti-PAX6 antibody (Table S7). PAX6-enriched peaks representing PAX6 binding sites on DNA were distributed across the genome in both PBS- and NMDA-injected retinas, predominantly within intronic and intergenic regions (Fig. 5G and Table S8). This suggests a broad recruitment of PAX6 to various genomic sites under both conditions, with a total of 7806 peaks identified. Despite the widespread presence of PAX6 across the genome, there was no significant difference in PAX6 binding at transcription start sites (TSS) between the NMDA- and PBS-treated groups (Fig. 5H). Overall, these results indicate that the presence of PAX6 at the TSS is consistent, regardless of NMDA treatment. Furthermore, we integrated the ChIP-Seq data with DEGs identified in NMDA-treated retinas. Importantly, approximately 28.6% (551 genes) of the upregulated target genes, which were influenced by both PAX6 and JNK3, overlapped with PAX6 ChIP-Seq peaks in NMDA-injected retinas (Fig. 5I). Additionally, this subset of genes was significantly enriched in pathways related to the apoptotic process, neuron apoptotic process, and apoptotic mitochondrial change (Fig. 5J). Compared to PBS-treated retinas, the binding of PAX6 at specific pro-apoptotic gene loci was markedly increased following NMDA treatment, although the global occupancy of PAX6 across the genome remained largely unchanged (Fig. 5K). Collectively, these findings suggest that NMDA treatment selectively enhanced PAX6 binding at apoptosis related genes. In particular, PAX6 was enriched at the promoters of pro-apoptotic genes such as *Bax* and *Gadd45a*, which were upregulated under NMDA-induced excitotoxic conditions (Fig. S6C). To validate the ChIP-seq findings, we performed ChIP-qPCR on a selected subset of target genes. Both PAX6 and JNK3 showed strong recruitment to the promoter regions of apoptotic genes in NMDA-treated retinas (Fig. 5L and Fig. S6D). In contrast, the recruitment of JNK3 to the promoters of apoptotic genes was significantly reduced in sh*Pax6*-GFP AAV2.7m8-infected retinas, suggesting that PAX6 is crucial for JNK3 recruitment to these sites. Overall, these results establish that PAX6 and JNK3 functionally cooperate to regulate the transcription of apoptotic genes via coordinated chromatin binding. This mechanistic interaction provides a direct link between extracellular stress signaling and transcriptional activation of neurodegenerative programs, reinforcing the therapeutic potential of targeting the PAX6–JNK3 axis to protect RGCs in retinal injury and glaucoma.

### PAX6 and JNK3 synergistically promote RGC apoptosis in excitotoxic retina

**Fig. 6:**
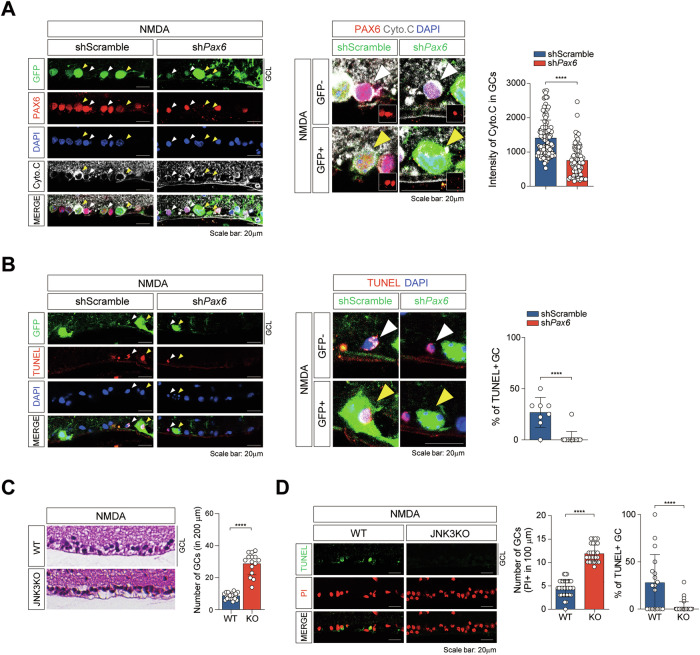
Knockdown or genetic deletion of PAX6 or JNK3 protects RGCs from excitotoxic apoptosis. **A** Immunostaining of NMDA-injected B6J WT mouse retinas with cytochrome c (white), PAX6 (red) antibodies, and DAPI (blue) following subretinal injection of shScramble-GFP AAV2.7m8 or sh*Pax6*-GFP AAV2.7m8. Higher magnification images show cytochrome c (red) expression. The intensity of cytochrome c in GC was quantified using ImageJ (right); Scale bar: 20 μm. **B** Apoptotic cells in NMDA-injected B6J WT mouse retinas were detected using TUNEL assay (BrdU+ cells) following subretinal injection of shScramble-GFP AAV2.7m8 or sh*Pax6*-GFP AAV2.7m8. Higher magnification images show BrdU (red) staining in ganglion cells. The percentage of BrdU-positive cones was quantified using ImageJ (right); scale bar: 20 μm. **C** NMDA-injected JNK3 KO mouse retinas (left) were stained with DAPI and H&E; scale bar: 20 μm. Quantification of the number of GC on 200 μm in NMDA-injected JNK3 KO or WT mouse retinas (right). **D** Apoptotic cells in NMDA-injected JNK3 KO or WT mouse retinas were detected using TUNEL assay (BrdU+ cells); scale bar: 20 μm. The number of GC and percentage of BrdU-positive GC is quantified (right) using ImageJ. Each quantification graph includes *n* = 10 per group, representing biologically independent retinas from three different animals. All error bars represent SEM. *P* values obtained based on Student’s *t*-test. **** *P* < 0.0001.

To assess the functional role of PAX6 in NMDA-induced RGCs apoptosis, we performed cytochrome c immunostaining in retinas transduced with AAV2.7m8-sh*Pax6*-GFP. In WT mice injected with control AAV (shScramble-GFP), NMDA exposure led to a substantial loss of RGCs and pronounced cytochrome c accumulation in the ganglion cell layer. In contrast, retinas with PAX6 knockdown maintained higher RGC density and exhibited minimal cytochrome c expression, indicating reduced mitochondrial apoptotic activity (Fig. 6A and Fig. S7A, B). To further assess the role of PAX6 in RGC apoptosis, we conducted a TUNEL assay on sh*Pax6*-GFP AAV2.7m8-treated retinas from 2-month-old mice. Notably, there was a significant increase in TUNEL-positive RGCs in the retinas of WT mice (shScramble-GFP AAV2.7m8-injected) following NMDA-induced excitotoxicity, indicating increased apoptosis (Fig. 6B and Fig. S7C). In contrast, there was no increase in TUNEL-positive RGCs in *Pax6*-deficient retinas (sh*Pax6*-GFP AAV2.7m8-infected) following NMDA-induced excitotoxicity (Fig. 6B and Fig. S7C). These results establish that PAX6 acts as a pro-apoptotic regulator in RGCs and is functionally required for the execution of NMDA-induced cell death. Considering the roles of JNK2 and JNK3 in promoting apoptosis in neurodegenerative diseases [23, 39], we examined whether JNK3 contributes to NMDA-induced RGC death. Importantly, JNK3 KO retinas exhibited a higher number of RGCs following NMDA injection than did retinas from WT mice (Fig. 6C), indicating that JNK3 inhibition protects against NMDA-induced RGC loss. To further confirm the role of JNK3 in RGC apoptosis, we performed a TUNEL assay on JNK3 KO retinas. The number of TUNEL-positive cells, which indicates proliferating or recently divided cells, significantly decreased in NMDA-injected JNK3 KO retinas, confirming reduced apoptosis in the absence of JNK3 (Fig. 6D). Together, these results demonstrate that both PAX6 and JNK3 are essential mediators of NMDA-induced retinal injury, acting in concert to drive apoptosis in ganglion cells. The parallel protective effects observed in PAX6 knockdown and JNK3-deficient retinas suggest a synergistic interaction between these two factors, consistent with their physical association and co-occupancy at pro-apoptotic gene loci. This mechanistic and functional evidence collectively establishes the JNK3–PAX6 transcriptional axis as a central driver of retinal neurodegeneration and highlights its potential as a gene-based therapeutic target in glaucoma.

## Discussion

**Fig. 7:**
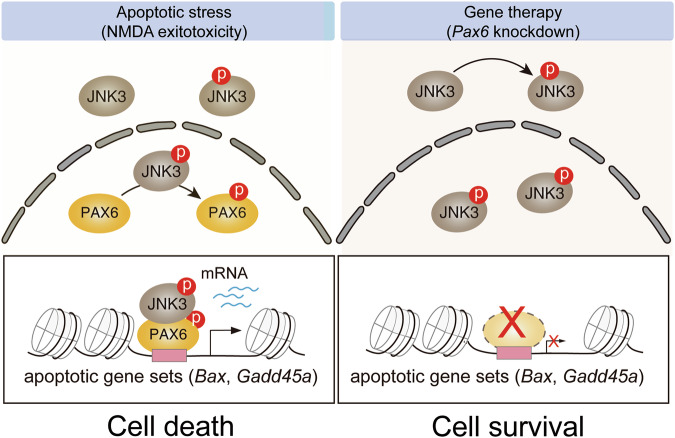
A schematic model illustrating the JNK3–PAX6 axis as a phosphorylation-dependent transcriptional mechanism driving RGC apoptosis. NMDA-induced activation of JNK3 phosphorylates PAX6, promoting its chromatin binding and transcription of pro-apoptotic genes (*Bax* and *Gadd45a*), leading to RGC death (left). AAV-sh*Pax6* gene therapy blocks this process, suppressing apoptotic gene activation and promoting RGC survival (right).

Glaucoma, a leading cause of irreversible blindness, is characterized by progressive degeneration of RGCs [2]. Although IOP reduction remains the primary therapeutic strategy, many patients continue to lose vision despite adequate IOP control, suggesting the involvement of IOP-independent neurodegenerative mechanisms. In this study, we identify a stress-activated transcriptional mechanism involving the JNK3-PAX6 axis that links excitotoxic injury to pro-apoptotic gene activation in RGCs (Fig. 7). Our results show that JNK3 phosphorylates PAX6 in response to NMDA-induced stress, facilitating its recruitment to chromatin and subsequent transcriptional activation of apoptotic genes.

Although classically defined as a developmental regulator, PAX6 expression persists into adulthood, contributing to axon guidance, retinal homeostasis, and postmitotic neuronal functions in the inner retina [40, 41]. Under pathological conditions, PAX6 is implicated in pro-apoptotic responses, including its upregulation in glaucoma models and promotion of cell death in the corneal epithelium [42, 43], suggesting a dual role in maintenance and degeneration. Consistent with this, our scRNA-seq analyses confirmed sustained PAX6 expression in mature RGCs (Fig. 1). Functionally, PAX6 knockdown using intravitreal sh*Pax6*-GFP AAV2.7m8 significantly reduced NMDA-induced RGC apoptosis, as evidenced by decreased cytochrome c release and fewer BrdU-positive nuclei (Fig. 6). Transcriptomic profiling revealed downregulation of pro-apoptotic genes, including Bax and Gadd45a, following PAX6 silencing (Figs. 2 and 5) [44, 45]. These findings demonstrate that PAX6 is not only retained in adult RGCs but also becomes transcriptionally active under pathological stress, where it contributes directly to the activation of apoptotic gene networks.

Phosphorylation is a major regulatory mechanism for transcription factor function. Although previous studies identified ERK, HIPK2, and p38 as PAX6 kinases [27, 28], our findings highlight JNK3 as the primary kinase mediating PAX6 phosphorylation under excitotoxic stress. Upon activation, JNK3 translocates to the nucleus, interacts with PAX6, and promotes its phosphorylation. This modification enhances PAX6 binding to apoptotic gene promoters, as confirmed by ChIP-seq and ChIP-qPCR analyses. Notably, PAX6 phosphorylation was absent in JNK3-deficient retinas, supporting a model in which JNK3 functions upstream of PAX6 to regulate stress-induced transcriptional responses. Although JNK2 is expressed in retinal neurons including RGCs, our IHC analysis showed minimal co-localization between JNK2 and PAX6 under NMDA-induced stress (Fig. S4G), justifying our focus on JNK3. Nonetheless, biochemical kinase assays under high enzyme-to-substrate conditions may reveal latent PAX6 kinase activity of JNK2, warranting further investigation in broader pathological contexts. Collectively, our findings delineate a stepwise mechanism whereby excitotoxic stress activates JNK3, which phosphorylates PAX6, thereby reprogramming transcription in favor of apoptosis (Figs. 3–5). This reveals how a developmental transcription factor can be repurposed in adult neurons under pathological conditions, with PAX6 phosphorylation linking stress-activated kinases to the transcriptional machinery of neuronal cell death. While our analyses strongly support a RGC-centered role of the JNK3-PAX6 axis, we acknowledge that displaced amacrine cells in the GCL may also contribute. Our scRNA-seq integration and amacrine cell and RGC marker-based immunostaining support persistent PAX6 expression and stress-dependent JNK3 interaction primarily in RGCs, yet bulk tissue analysis cannot fully exclude contributions from displaced amacrine cells. Future single-cell or spatial transcriptomic approaches will be essential to definitively resolve cell-type specificity of this pathway. Nonetheless, our current data highlight that the predominant pathological impact of JNK3-mediated PAX6 phosphorylation arises within RGCs, consistent with their selective vulnerability in glaucoma.

From a therapeutic standpoint, targeting transcriptional regulators such as PAX6 offers an upstream strategy to modulate neurodegenerative pathways. AAV-mediated PAX6 silencing provided robust neuroprotection in vivo, preserving both RGC morphology and transcriptional homeostasis. Given that RGC loss often progresses despite normalized IOP, gene regulatory interventions may complement, or even surpass, current pressure-lowering therapies [46]. Unlike downstream neuroprotective strategies, such as brimonidine or Bcl-xL overexpression, the JNK3-PAX6 axis operates at the level of transcriptional initiation. This upstream positioning enables mechanistic precision and allows for combination strategies. For example, NMDA receptor blockade could be paired with PAX6 silencing to inhibit both stress induction and downstream apoptotic signaling. Our findings also support the translational feasibility of this approach, as sh*Pax6*-GFP AAV2.7m8 effectively silenced PAX6 in RGCs and prevented excitotoxic damage. Similar neuroprotective effects have been reported with other AAV-based strategies, including CaMKIIα -driven axonal preservation and Bcl-xL-mediated survival [47, 48]. Nonetheless, further work is needed to address key challenges such as immunogenicity, long-term expression, and cell-type specificity. Non-viral platforms, such as lipid nanoparticles-mediated CRISPR interference, may offer additional precision and safety advantages [49]. Although our study focused on acute NMDA-induced excitotoxicity, JNK signaling is also implicated in chronic RGC degeneration. Therefore, the JNK3-PAX6 axis may be relevant in chronic glaucoma models such as DBA/2 J or microbead occlusion. Investigating this pathway in chronic settings will be essential to fully assess its translational potential.

Conclusively, our study identifies a stress-responsive transcriptional circuit in which JNK3-mediated phosphorylation of PAX6 drives apoptotic gene reprogramming in mature retina. This mechanism redefines the role of a canonical developmental transcription factor in adult neurodegeneration and reveals the PAX6–JNK3 axis as a critical mediator of RGC apoptosis. Most importantly, we demonstrate that targeted suppression of this axis via AAV-mediated gene silencing confers robust neuroprotection in vivo, offering a novel therapeutic paradigm for glaucoma (Fig. 7). As conventional IOP-lowering strategies remain insufficient to prevent vision loss, transcriptionally guided interventions targeting the JNK3–PAX6 pathway may serve as a complementary or even primary strategy for preserving retinal function in glaucomatous neurodegeneration.

## Supplementary information

Supplementary Table S1

Supplementary Table S2

Supplementary Table S3

Supplementary Table S4

Supplementary Table S5

Supplementary Table S6

Supplementary Table S7

Supplementary Table S8

Supplementary Table S9

Supplementary Table S10

SD Figure legends

SD Figures

Original data files

aj checklist

## Data Availability

Raw and processed RNA-Seq (Figs. 2 and 5) data has been deposited in Gene Expression Omnibus under accession code GSE275656. ChIP-Seq (Fig. 5) data has been deposited in Gene Expression Omnibus under accession code GSE275655. The human and mouse scRNA-seq (Fig. 1) data are available at GEO Accession number GSE196235, GSE216694, and GSE243413.
