# Supplementary material for: Aberrant maintenance of developmental transcription factor PAX6 promotes neuronal cell death via JNK3 signaling
Source: Cell Death Dis. 2026 Jan 29;17(1):161. doi: 10.1038/s41419-026-08417-6 (PMC12876059; doi:10.1038/s41419-026-08417-6)
Supplement: Supplementary file 11 — SD Figure legends [file 41419_2026_8417_MOESM11_ESM.docx]

**SUPPLEMENTARY FIGURE LEGENDS**

**Supplementary Fig. 1 Single-cell transcriptome analysis of mouse and human retinas. A, B** Feature expression heatmap showing retinal cell type specific markers in mouse (10 groups) (**A**) and human (13 groups) (**B**) retinas. **C** Co-immunostaining of PAX6 (red) with Calb1 and ChAT (amacrine marker) and Brn3a (RGC marker) in the GCL. Nuclei are counterstained with DAPI (white); scale bar: 50 μm.

**Supplementary Fig. 2 Establishment of glaucoma-like excitotoxic model.** **A** Schematic diagram of mouse eye dissection and retinal isolation for histological analysis. **B** Whole mount retinas of PBS- or NMDA-injected mouse stained with DAPI (blue). **C** Apoptotic cells in the INL of PBS- and NMDA-injected mice retinas were detected using TUNEL assay (BrdU^+^ cells) with BrdU (green) and PI (red) staining; scale bar: 20 μm. **D** Immunostaining of PBS- and NMDA-injected B6J wild-type retinas with cytochrome c (green) and DAPI (blue); scale bar: 20 μm. **E** Representative immunofluorescence images showing co-staining of cytochrome c (red) with the amacrine cell marker ChAT (green) and DAPI (white) in PBS- or NMDA-treated retinas. scale bar: 50 μm. **F** Quantification of cell subpopulations presented as stacked percentages (upper panel) and absolute cell numbers (lower panel). Each quantification graph includes n = 3 per group, representing biologically independent retinas from three different animals. All error bars represent SEM.

**Supplementary Fig. 3 PAX6 knockdown suppresses NMDA-induced pro-apoptotic transcriptional programs in RGCs. A**, **B** Outline of experimental design (**A**) and AAV reporter cassettes for knockdown of PAX6 (**B**). **C** Immunostaining of B6J wild-type mouse retinas with PAX6 following subretinal injection of control shScramble-GFP AAV2.7m8 or sh*Pax6*-GFP AAV2.7m8. Quantification of the intensity of PAX6-positive ganglion cells (right); scale bar: 20 μm. Each quantification graph includes n = 3 per group, representing biologically independent retinas from three different animals. All error bars represent SEM. *P* values were obtained based on Student's *t*-test. *** *P* < 0.001. **D, E** Volcano plots showing transcriptomic changes in B6J (**D**) and sh*Pax6*-GFP AAV2.7m8-infected retinas (**E**) treated with PBS or NMDA. Significantly upregulated transcripts (log2FC > 1, P < 0.05) are shown in red, and significantly downregulated transcripts (log2FC < -1, P < 0.05) are shown in blue. Transcripts not meeting these thresholds are displayed in gray. **F** Scatter-line plot showing WCSS (Within-Cluster Sum of Squares) score for k-means clustering on RNA-seq data. Red dashed line indicates the optimal point for k = 4 clusters. **G** Heatmap showing DEGs (in PBS- or NMDA-injected) in control (B6J) and sh*Pax6*-GFP AAV2.7m8-infected (sh*Pax6*) mouse retinas mapped to apoptotic processes.

**Supplementary Fig. 4 PAX6 interacts with JNK3 in the retina of mice with NMDA-induced excitotoxicity.** **A** Immunostaining of B6J wild-type mouse retinas injected with PBS and NMDA with PAX6. Quantification of the intensity of PAX6-positive ganglion cells (right); scale bar: 20 μm. Each quantification graph was based on data from five biologically independent retinas from three different animals. All error bars represent SEM. *P* values were obtained based on Student's *t*-test. n.s., not significant. **B** Profiles of PAX6 binding to the kinases in the MAPK phosphorylation antibody array. **C**, **D** t-SNE visualization of the expression patterns of JNK family (*Mapk8*, *Mapk9*, and *Mapk10*) and kinases (*Map2k1*, *Map2k6*, and *Rps6ka1*) in mouse (**C**) and human (**D**) retinas. **E** Feature expression heatmap showing the JNK family and kinases with retinal cell type specific markers in mouse (10 groups) and human (13 groups) retinas. **F** Immunoblot showing co-immunoprecipitation of endogenous JNK1, JNK2, and JNK3 with PAX6 in mice retinas. The blot was first probed with anti-PAX6 (top panel) and subsequently stripped and re-probed with anti-JNK (bottom panel). The same membrane was used for both probings. **G** Immunostaining of mouse retinas treated with PBS or NMDA, using antibodies against PAX6 (red) and JNK1, JNK2, or JNK3 (green). White arrows indicate co-localization between PAX6 and the respective JNK isoforms. **H** Immunostaining of NMDA-injected B6J wild-type mouse retinas with PAX6 (white) and JNK3 (red) antibodies and DAPI (blue) following subretinal injection of sh*Pax6*-GFP AAV2.7m8.

**Supplementary Fig. 5 JNK3 phosphorylates PAX6 *in vitro* and *in vivo*. A** Purified GST-PAX6 and GST-JNK3 were incubated with [γ-^32^P] ATP isotope or cold ATP for 30 min at 30 ℃. Reaction mixtures were separated using SDS-PAGE and analyzed via an autoradiography, with GST-NRL and GST-c-Jun as positive controls and GST alone as a negative control. **B** Immunoprecipitation and immunoblot showing endogenous phosphorylation of JNK3 in PBS- and NMDA-treated mouse retinas. **C** *In* *vitro* kinase assay using GST-PAX6 with increasing concentrations of GST-JNK3. Representative autoradiography images for phosphorylated proteins (upper panel) or Coomassie staining for total proteins (lower panel). **D** Kinase assay as described in panel (**A**), GST-PAX6 and GST-JNK3 were pretreated with increasing JNK inhibitor SP600125. Representative autoradiography images of phosphorylated proteins (upper panel) or Coomassie staining for total proteins (lower panel).

**Supplementary Fig. 6 PAX6 and JNK3 directly regulate pro-apoptotic functions in the retina of mice with NMDA-induced excitotoxicity.** **A** Correlation matrix of Pearson correlation coefficient values for each indicated sample set. Shades of red indicate an increasing positive correlation coefficient. **B** Volcano plot showing transcriptomic changes in JNK3 KO retinas treated with PBS or NMDA. Significantly upregulated (red) and downregulated (blue) transcripts were identified using thresholds of (abs(log_2_FC) > 1.0, *P* < 0.05). **C** ChIP-seq and RNA-seq tracks of PAX6 in PBS- or NMDA-treated mouse retinas at the selected target genes loci (*Bax*, *Gadd45a*, *Ing4*, *Dedd2*, *Bad,* and *Pdcd2)*. RNA-seq tracks represent gene expression under the respective treatment conditions. **D** Recruitment and presence of PAX6 and JNK3 at *Ing4*, *Dedd2*, *Bad,* and *Pdcd2* promoter regions were validated using ChIP-qPCR. Error bars show mean ± SE. *P* values were obtained using Student's *t*-test. **** *P* value < 0.0001, ** *P* value < 0.01, * *P* value < 0.05, n.s., not significant.

**Supplementary Fig. 7 PAX6 and JNK3 inhibitions protect against ganglion cell death.** **A** Quantification of immunostaining intensity in the nucleus of NMDA-injected B6J wild-type mouse retinas following subretinal injection of shScramble-GFP AAV2.7m8 or sh*Pax6*-GFP AAV2.7m8. **B** Immunostaining of PBS- and NMDA-injected B6J wild-type mouse retinas with cytochrome c (white) and PAX6 (red) antibodies and DAPI (blue) following subretinal injection of shScramble-GFP AAV2.7m8 or sh*Pax6*-GFP AAV2.7m8. High magnification images showing cytochrome c (red) expression, and the percentage of cytochrome c is quantified (right); scale bar: 20 μm. **C** Apoptotic cells were detected in PBS- and NMDA-injected B6J wild-type mouse retinas using TUNEL assay (BrdU+ cells) following subretinal injection of shScramble-GFP AAV2.7m8 or sh*Pax6*-GFP AAV2.7m8. High magnification images showing BrdU (red) staining in ganglion cells, and the percentage of BrdU-positive cells is quantified (right); scale bar: 20 μm.
