# Supplementary material for: Aberrant maintenance of developmental transcription factor PAX6 promotes neuronal cell death via JNK3 signaling
Source: Cell Death Dis. 2026 Jan 29;17(1):161. doi: 10.1038/s41419-026-08417-6 (PMC12876059; doi:10.1038/s41419-026-08417-6)

**Supplementary Fig. 1**


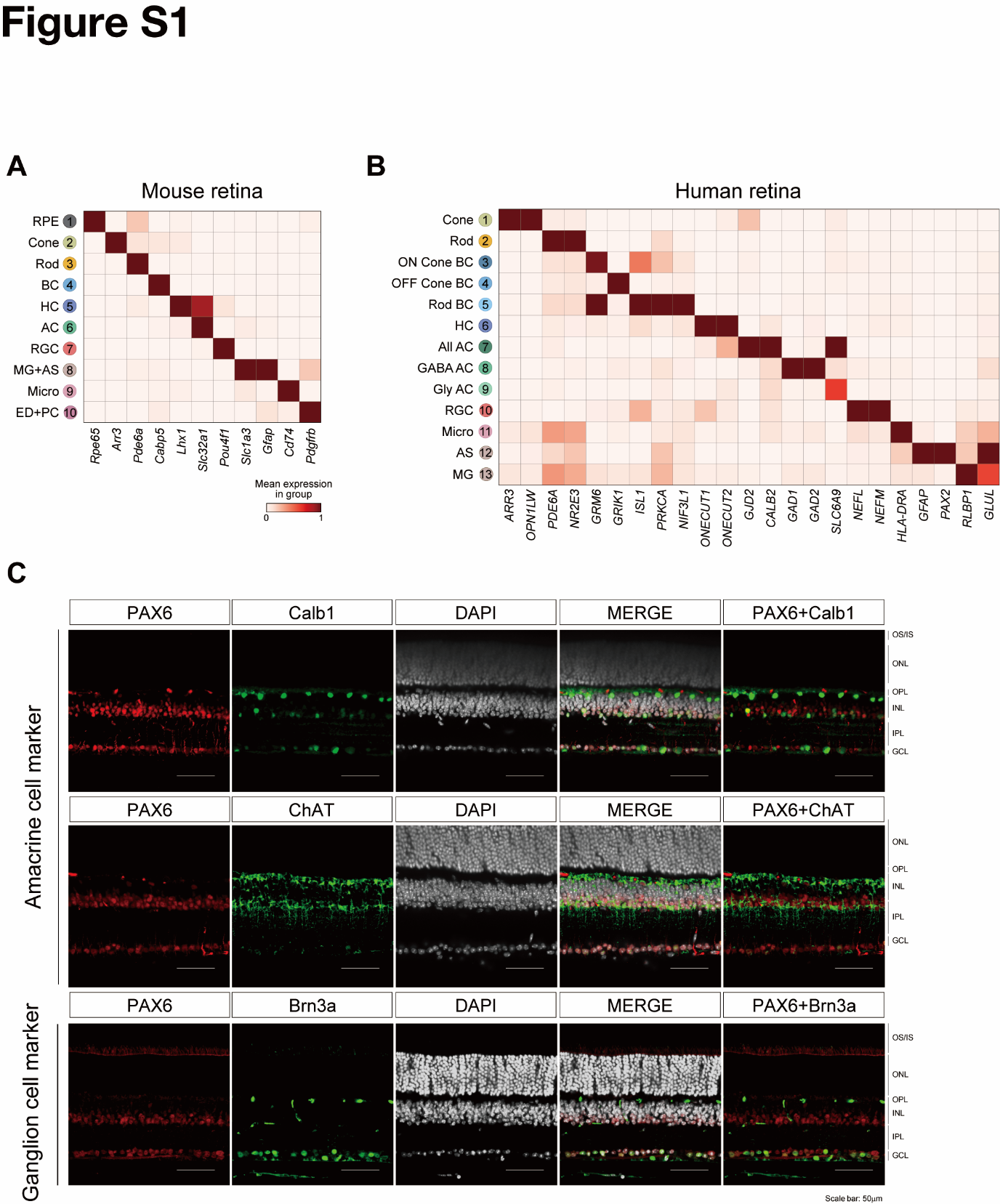


**Supplementary Fig. 2**


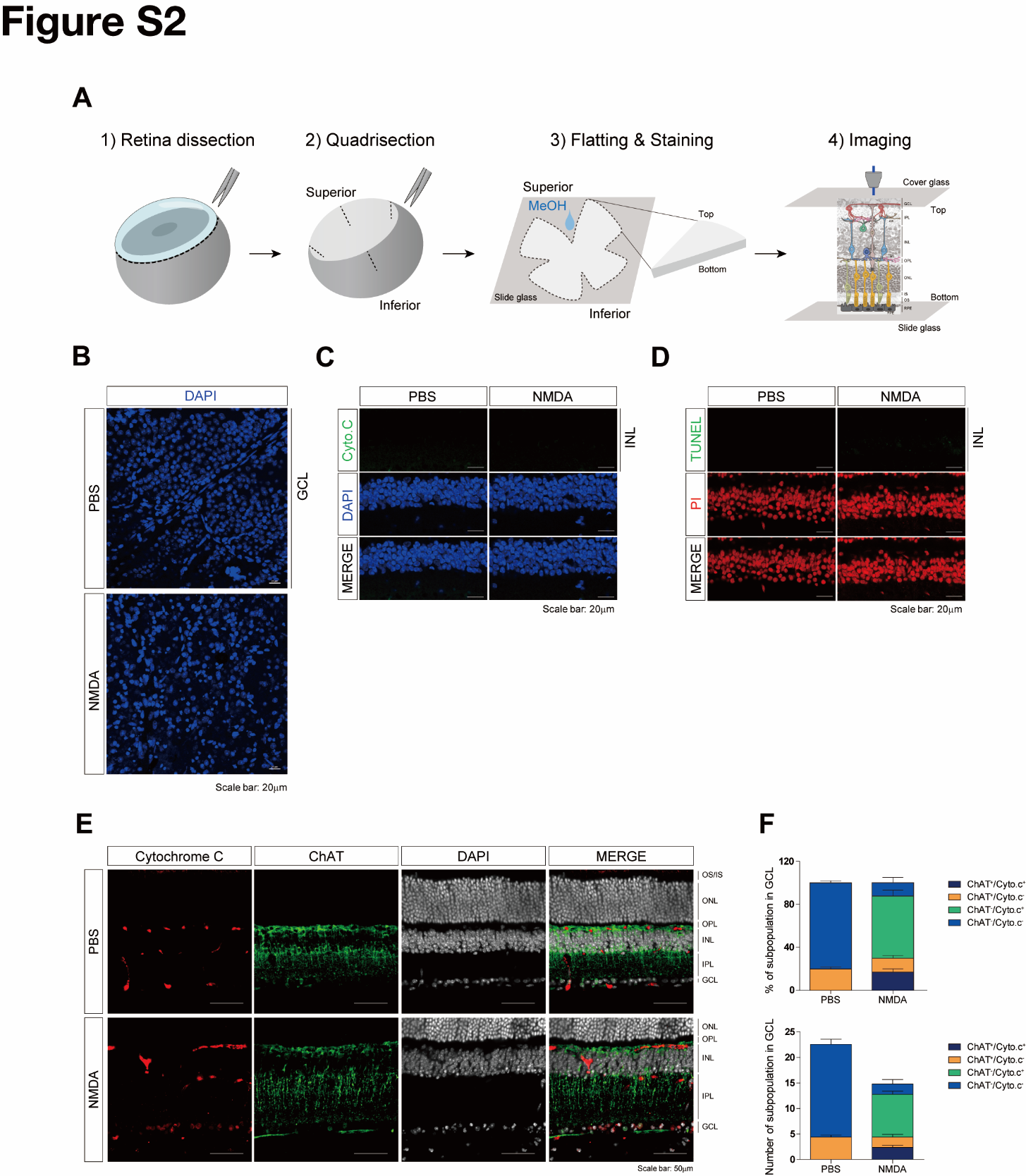


**Supplementary Fig. 3**


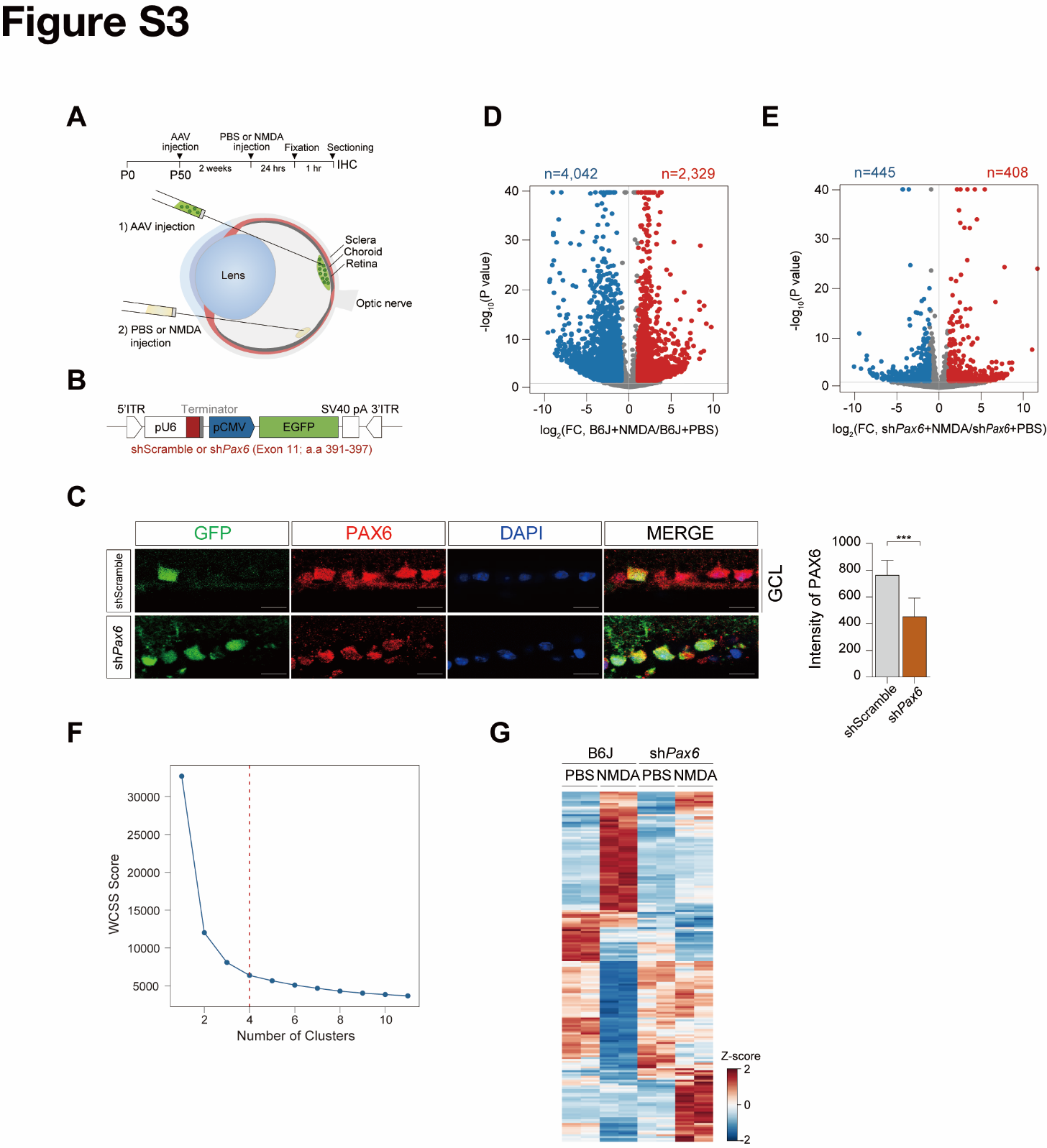


**Supplementary Fig. 4**


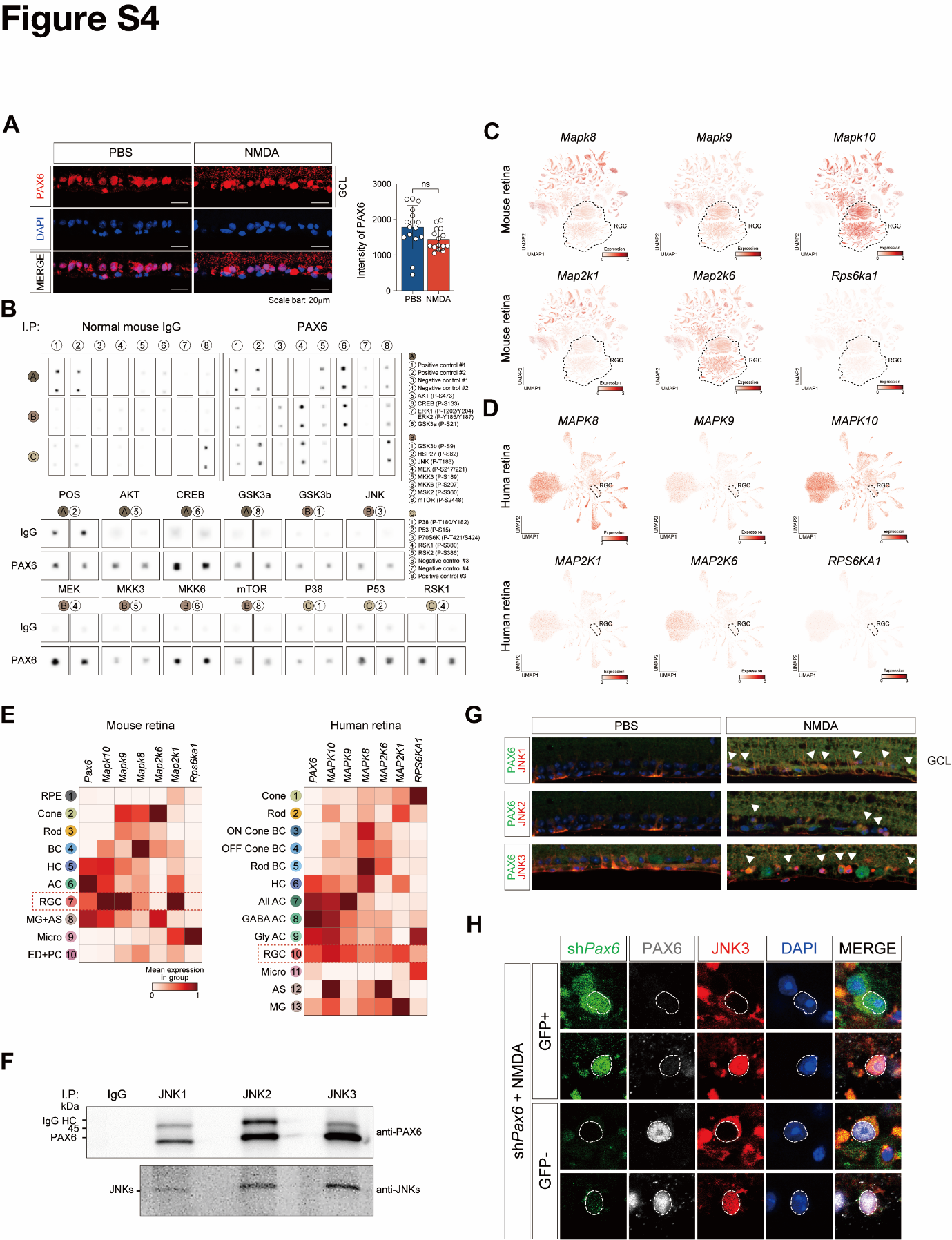


**Supplementary Fig. 5**

**
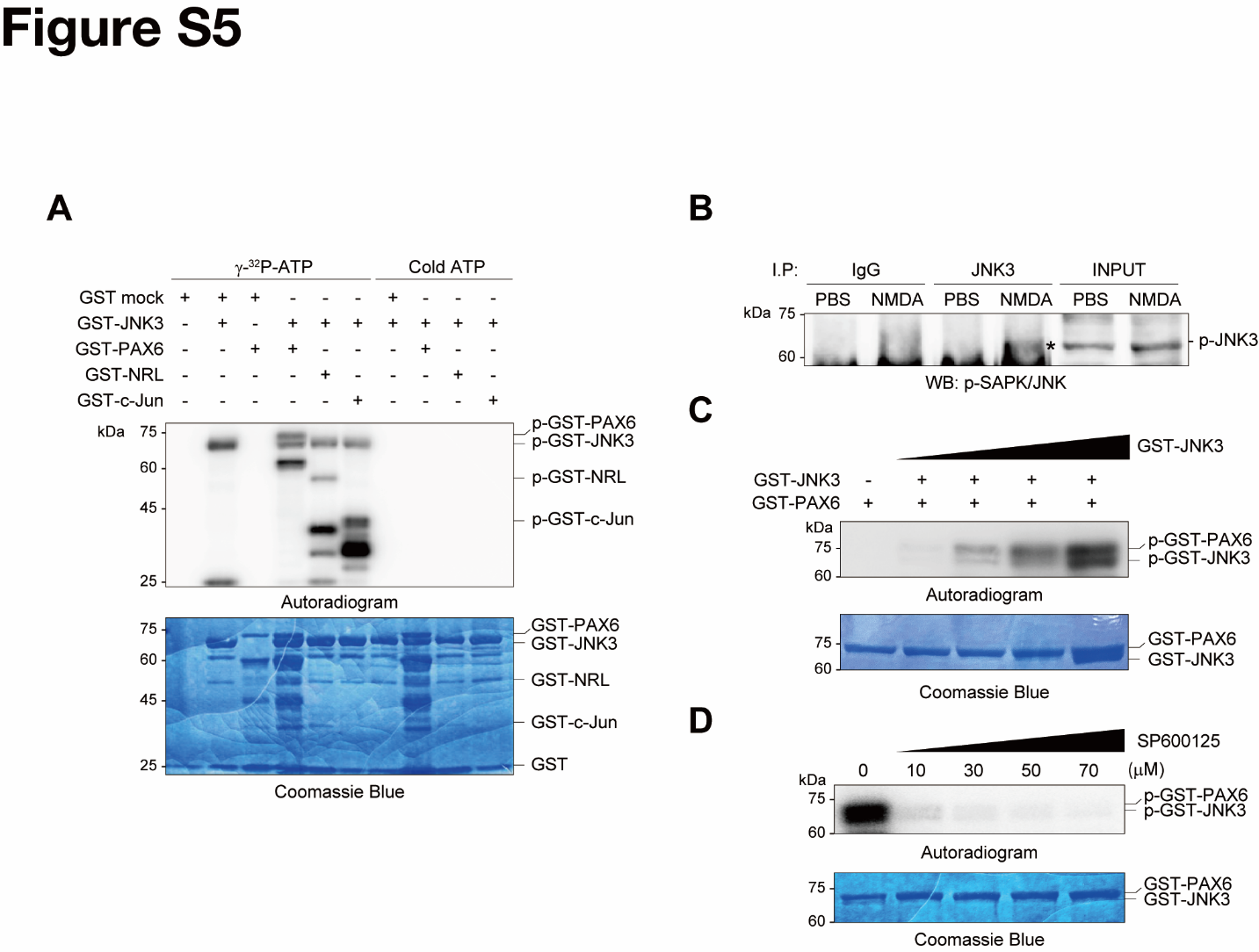
**

**Supplementary Fig. 6**


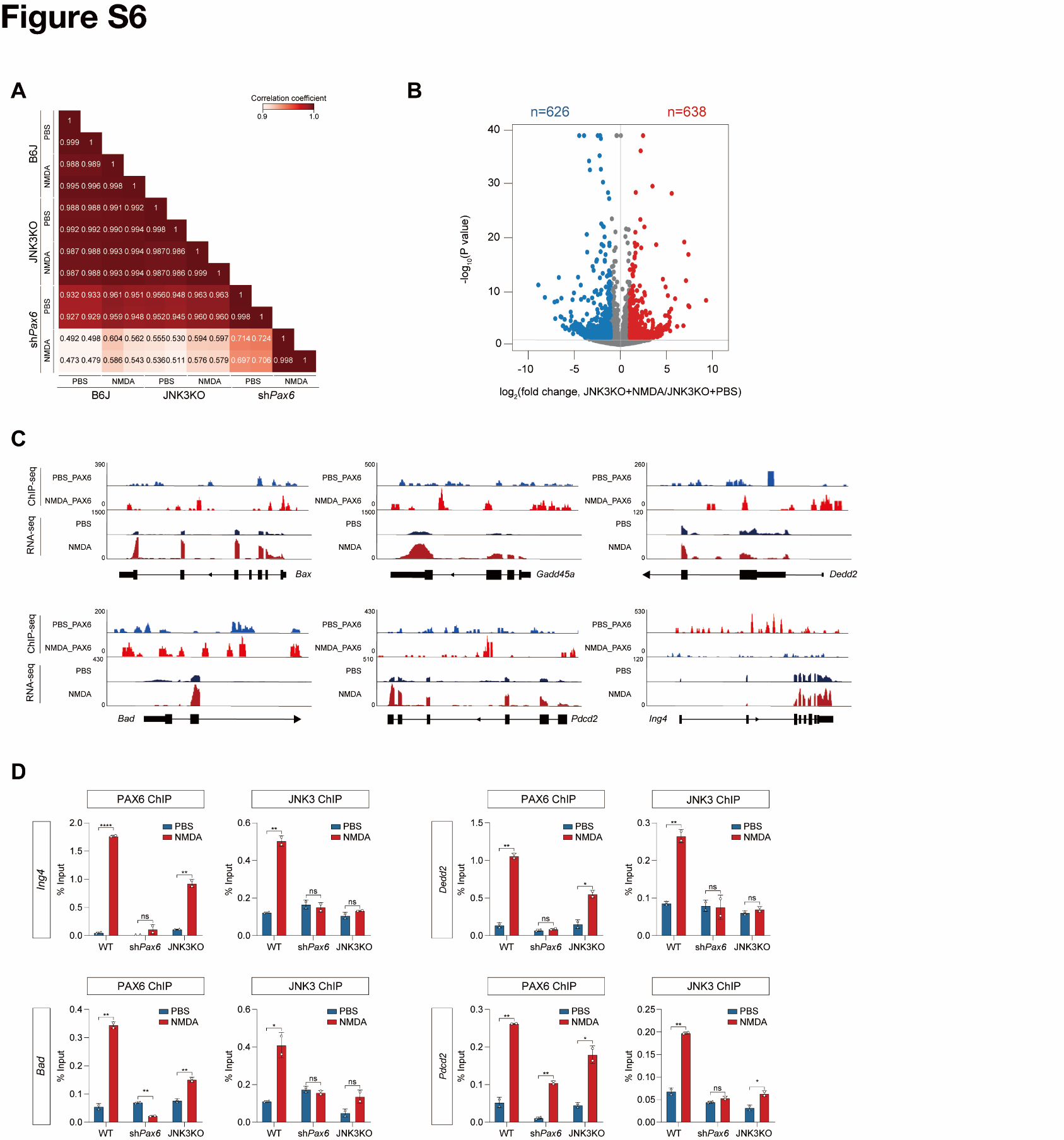


**Supplementary Fig. 7**


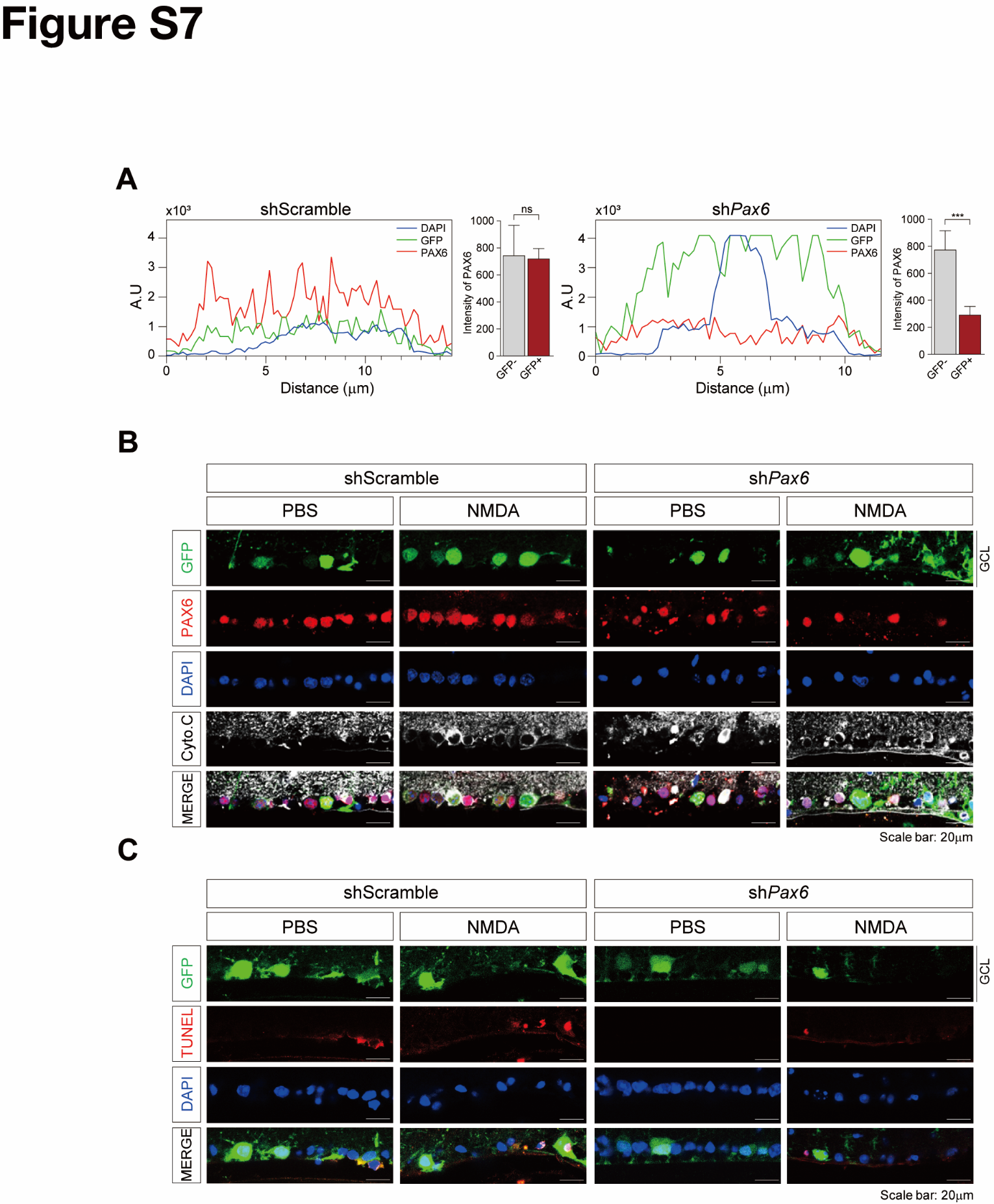

Supplement: Supplementary file 12 — SD Figures [file 41419_2026_8417_MOESM12_ESM.docx]
