# Supplementary material for: Aberrant maintenance of developmental transcription factor PAX6 promotes neuronal cell death via JNK3 signaling
Source: Cell Death Dis. 2026 Jan 29;17(1):161. doi: 10.1038/s41419-026-08417-6 (PMC12876059; doi:10.1038/s41419-026-08417-6)

Figure 3A

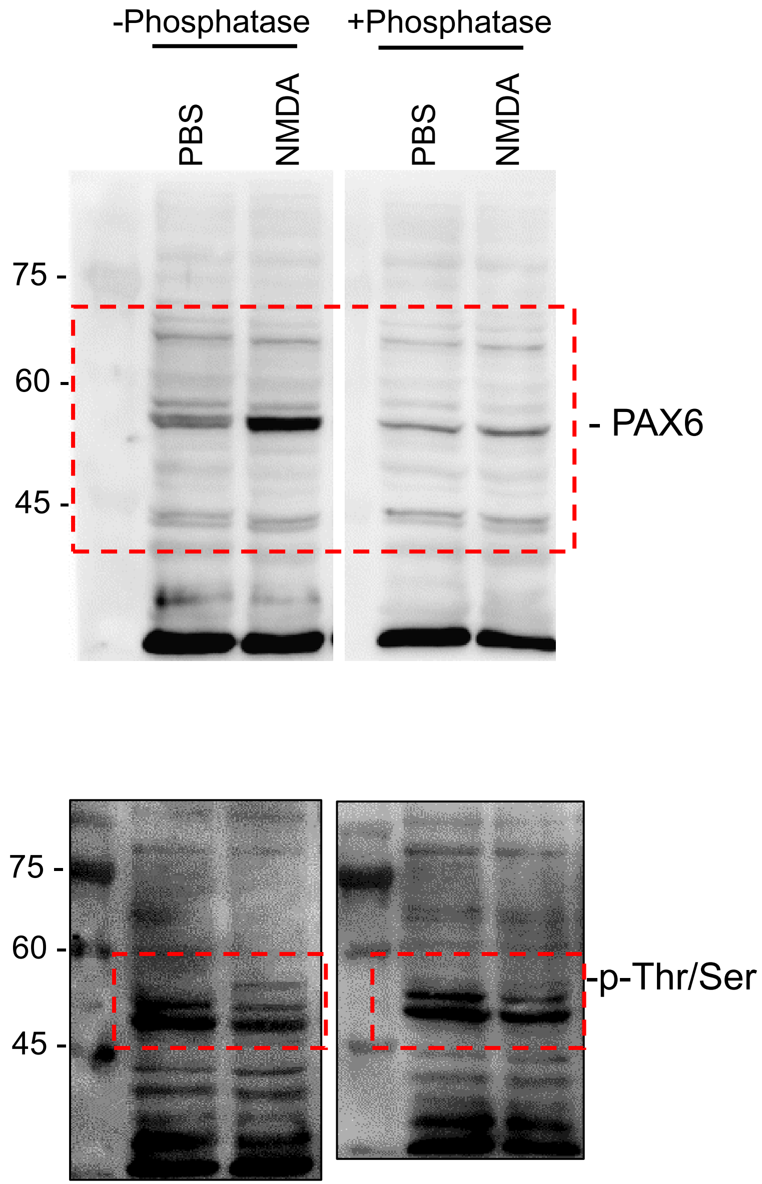
-Phosphatase
+Phosphatase
PBS
NMDA
PBS
NMDA
75 -
60 -
45 -
- PAX6
75 -
60 -
45 -
-p-Thr/Ser

Figure 3E

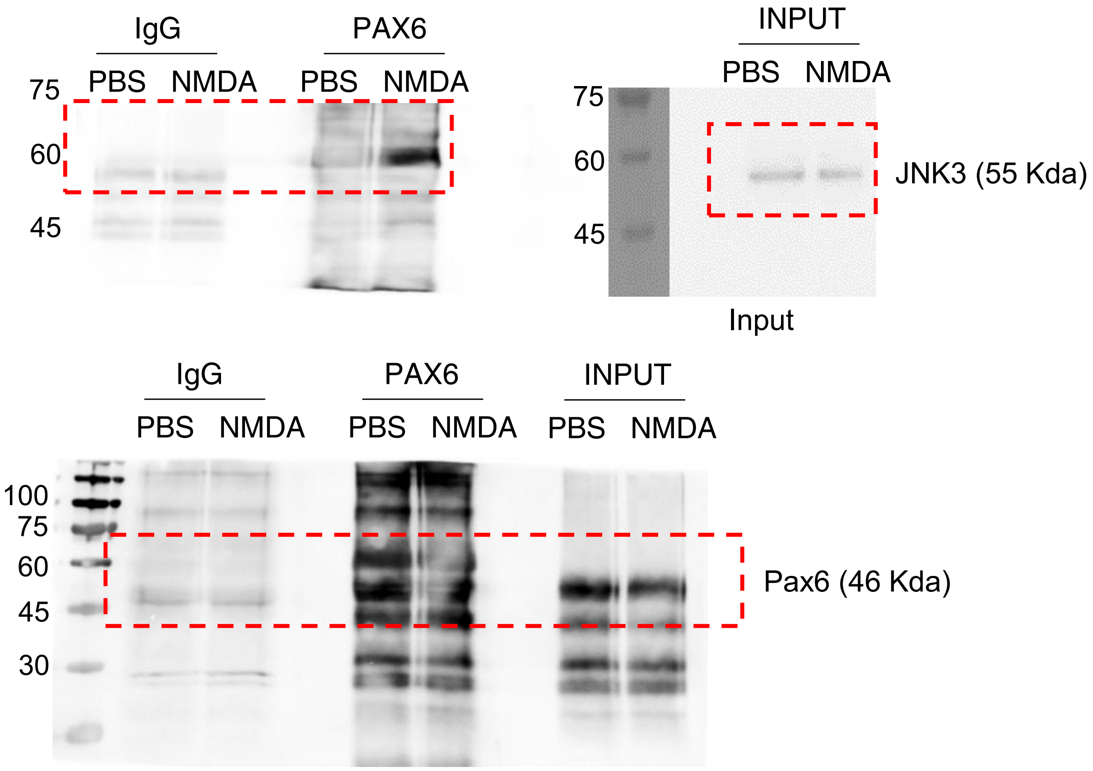
IgG
PAX6
INPUT
PBS
NMDA
PBS
NMDA
PBS
NMDA
75
60
45
75
60
45
JNK3 (55 Kda)
Input
IgG
PAX6
INPUT
PBS
NMDA
PBS
NMDA
PBS
NMDA
100
75
60
45
30
Pax6 (46 Kda)

Figure 4C

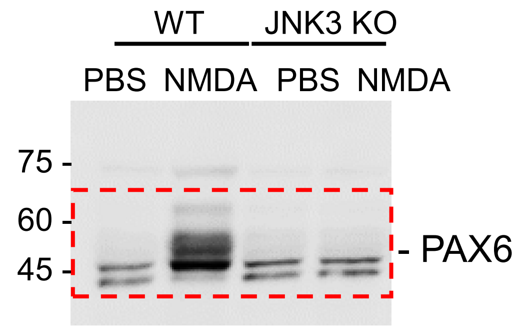

Figure 4D

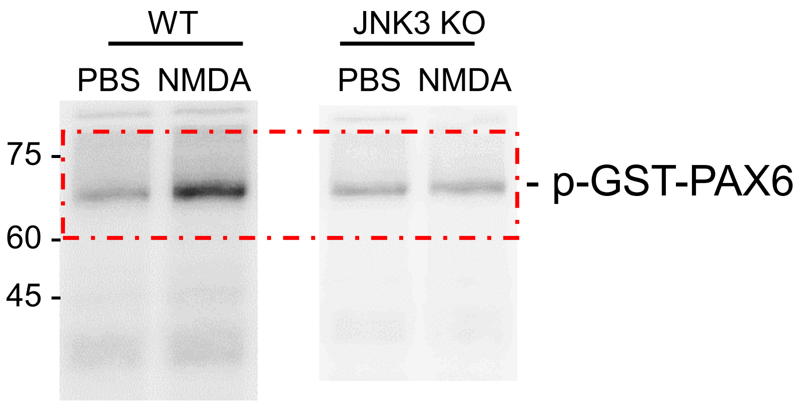

Supplementary Fig. 3F

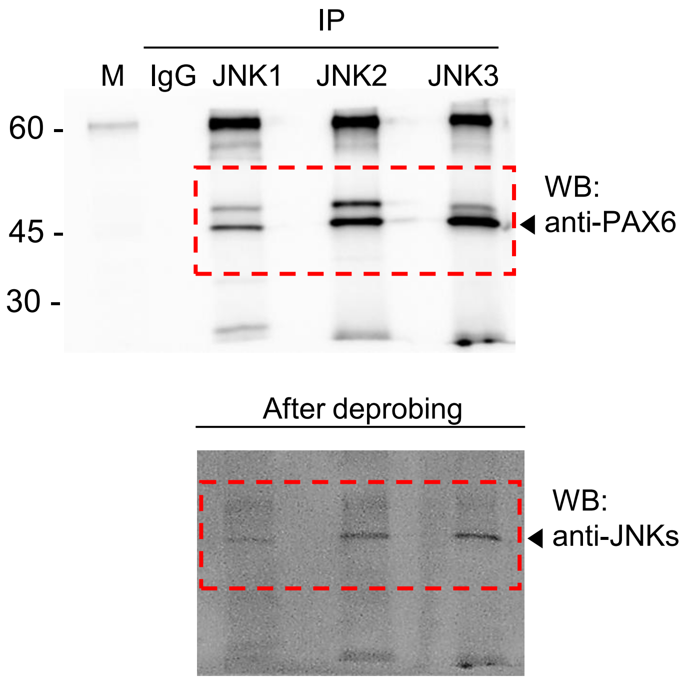

Supplementary Fig. 4B

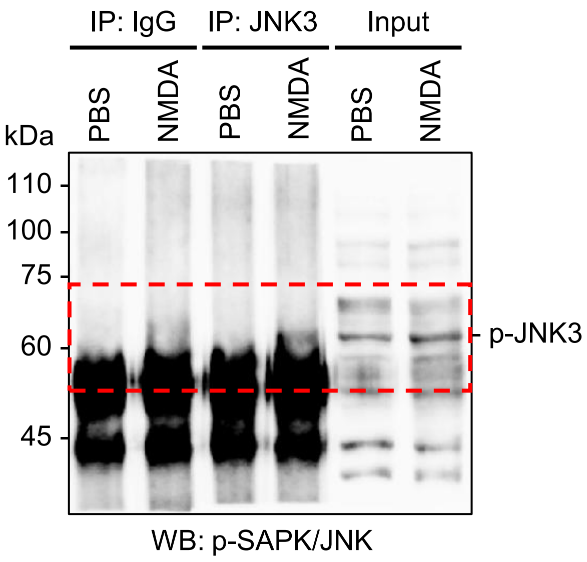

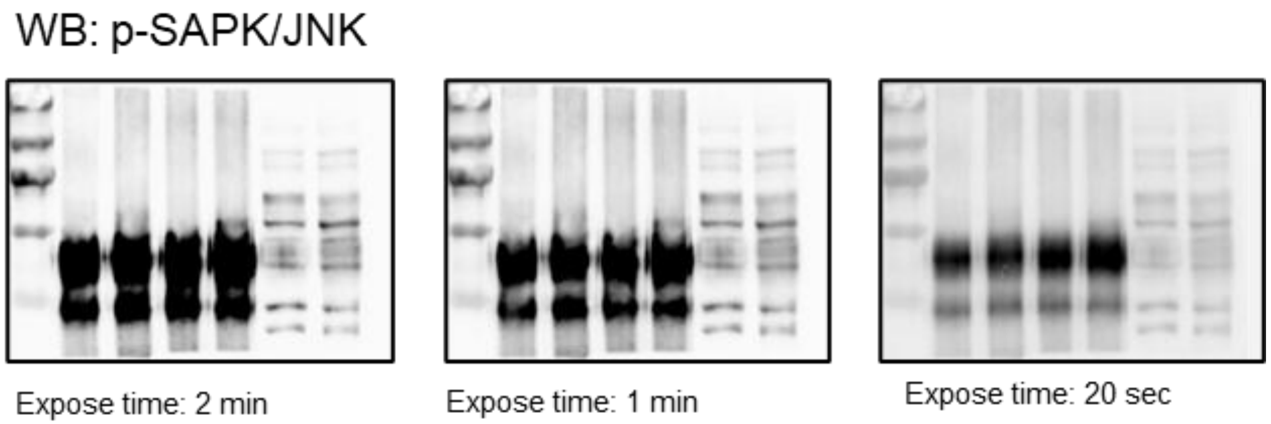

Supplement: Supplementary file 13 — Original data files [file 41419_2026_8417_MOESM13_ESM.pdf]
